# Causal associations between circulating metabolites and chronic kidney disease: a Mendelian randomization study

**DOI:** 10.1080/0886022X.2025.2498090

**Published:** 2025-04-29

**Authors:** Jie He, Lin Li, Hongjie Hu

**Affiliations:** Department of Radiology, Sir Run Run Shaw Hospital, Zhejiang University School of Medicine, Hangzhou, Zhejiang, P.R. China

**Keywords:** Chronic kidney disease, renal function, circulating metabolites, Mendelian randomization, glycine, N-acetylornithine

## Abstract

**Background:**

Circulating metabolites have been associated with cross-sectional renal function in population-based research. Nevertheless, there is currently little proof to support the idea that metabolites either cause or prevent renal function. New treatment targets and ways to screen individuals with impaired renal function will be made possible *via* an in-depth analysis of the causal relationship between blood metabolites and renal function.

**Methods:**

We assessed the causal relationship between 452 serum metabolites and six renal phenotypes (CKD, rapid progression to CKD [CKDi25], rapid eGFR decline [CKD rapid3], dialysis, estimated glomerular filtration rate, and blood urea nitrogen) using univariate Mendelian randomization, primarily employing the inverse variance weighted method with robust sensitivity analyses. Heterogeneity and pleiotropy were examined *via* Cochrane’s Q test and MR-Egger regression, and statistical significance was adjusted using Bonferroni correction. To assess potential adverse effects of metabolite modulation, we conducted a phenome-wide Mendelian randomization analysis, followed by multivariate Mendelian randomization to adjust for confounders.

**Results:**

We identified glycine and N-acetylornithine as potential causal mediators of CKD and renal dysfunction. Notably, lowering glycine levels may increase the risk of cholelithiasis and cholecystitis, while reducing N-acetylornithine could have unintended effects on tinnitus.

**Conclusion:**

Glycine and N-acetylornithine represent promising therapeutic targets for CKD and renal function preservation, but their modulation requires careful risk-benefit assessment to avoid adverse effects.

## 1. Introduction

Impaired renal structure and function are the characteristic of chronic kidney disease (CKD), which is often diagnosed by measuring an estimated glomerular filtration rate (eGFR) of less than 60 mL/min per 1.73 m^2^ or by observing increased kidney damage markers over at least three months [1]. Approximately 10% of adults worldwide suffer from CKD, which results in 1.2 million deaths and 28 million years of lost life annually on average [2]. Managing the traditional CKD risk factors, such as obesity, diabetes, and hypertension, may assist in preventing CKD [3–5]. Nevertheless, the burden of CKD is anticipated to rise considerably more during the next several years, despite all efforts toward avoiding it [6,7]. This emphasizes how critical it is to enhance CKD prevention. As a result, it’s critical to learn more about the pathophysiological and molecular mechanisms behind the development of CKD and discover novel targets for treatment to mitigate the deterioration of renal function.

Metabonomics, the newest addition to the field of systems biology, offers a novel approach to investigating the possible mechanisms behind disease and discovering new targets for therapeutic intervention. Metabonomics provides an expanded understanding of the pathological network of diseases by taking into account the intimate connection between metabolites and genotypes as well as phenotypes [8,9]. The possibility of approving metabolite medication targets based on genetic data is twice that of other drug targets [10]. Moreover, a number of genome-wide association studies (GWAS) have made significant progress recently in identifying the genetic origins of integrated human metabolites [11,12]. Mendelian randomization (MR) analysis is a new analytical technique that uses genetic variation as a representative of exposure to assess the causal relationship between exposure and outcome without confusion or reversal. As a result, we can accurately identify new and safe drug targets for the prevention of CKD at the genetic and metabonomic levels.

Currently, MR design has been used to determine the possible causal association between a number of biomarkers and the kidney. For example, beta-trace protein (BTP) and indoleamine 2,3-dioxygenase (IDO) may be risk factors for CKD and renal function [13,14]. These initiatives, however, will not suffice to comprehend the pathophysiology of CKD. To extensively evaluate potential therapeutic targets for the prevention of CKD and the decline of renal function in the human metabolic group, we conducted an MR analysis. Before conducting clinical trials, the phenome-wide MR (Phe-MR) study may reveal potential side effects of therapeutic targets. Here, we used MR analysis to find potential therapeutic targets for six phenotypes associated with CKD and renal function, including CKD, CKDi25, CKD rapid3, dialysis, eGFR and blood urea nitrogen (BUN). Firstly, we searched for putative causative mediators of CKD and renal function using 452 circulating metabolites. The causative independence of metabolites to CKD and renal function was then assessed using multivariate Mendelian randomization (MVMR), which was utilized to adjust for confounding factors. In order to completely evaluate the clinical safety of the discovered metabolites as a target of intervention, we finally carried out a Phe-MR analysis to forecast possible side effects.

## 2. Materials and methods

### 2.1. MR design and data source

**Figure 1.**
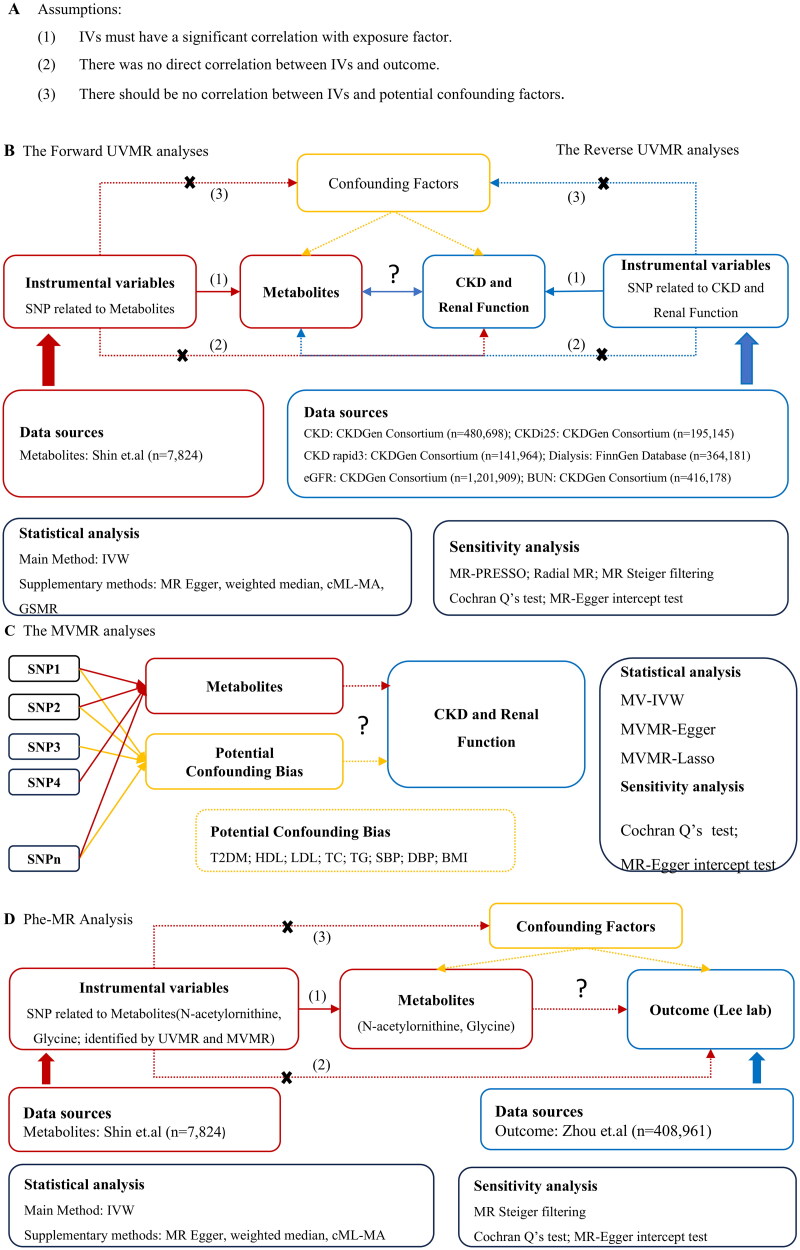
The outline of the methodology. (A) The assumptions for MR analysis. (B) The flow of UVMR analysis. (C) The flow of MVMR analysis. (D) The flow of Phe-MR analysis. Abbreviations: IVW: inverse variance weighted; MR: Mendelian randomization; MR-PRESSO: MR pleiotropy residual sum and outlier; GSMR: generalized summary data-based Mendelian randomization; IVs: instrumental variables; UVMR: univariate Mendelian randomization; MVMR: multivariate Mendelian randomization; Phe-MR: phenome-wide Mendelian randomization; CKD: chronic kidney disease; CKDi25: defined as the decrease of eGFR ≥ 25% of baseline accompanied by the progression from no CKD to CKD; CKD rapid3: eGFR decreases by more than 3 mL/min/1.73m^2^ per year; eGFR: estimated glomerular filtration rate; BUN: blood urea nitrogen; T2DM: type 2 diabetes; HDL: high-density lipoprotein cholesterol; LDL-C: low-density lipoprotein cholesterol; TC: total cholesterol; TG: triglyceride; SBP: systolic blood pressure; DBP: diastolic blood pressure; BMI: body mass index.

First, a two-sample univariate Mendelian randomization (UVMR) was employed to search for putative circulating metabolites associated with CKD and renal function and to thoroughly evaluate the bidirectional causal connection between the circulating metabolites and CKD as well as renal function. Second, MVMR analysis was then utilized to assess whether these putative circulating metabolites contributed to the development of CKD and renal function on their own. Third, in order to investigate possible side effects targeting identified metabolites, we performed a Phe-MR study of 677 non-kidney illnesses. The outline of the methodology is represented in Figure 1. Summary-level data based on GWAS carried out among participants with mainly European ancestry were used in this MR analysis (Table 1).

**Table 1.** Detailed information for the GWAS data.

Phenotype	Consortium	Author	Sample size	Cases/controls	PMID	Year	Population
Metabolites	Meta-analysis	Shin et al	7,824	NA	24816252	2014	European
CKD	CKDGen Consortium	Wuttke et al	480,698	41,395/439,303	31152163	2019	European
CKDi25	CKDGen Consortium	Gorski et al	195,145	19,901/175,244	33137338	2021	European
CKD rapid3	CKDGen Consortium	Gorski et al	141,964	34,874/107,090	33137338	2021	European
Dialysis	FinnGen	Kurki et al	364,181	1004/363,177	36653562	2023	European
eGFR	CKDGen Consortium	Stanzick et al	1,201,909	NA	34272381	2021	European
BUN	CKDGen Consortium	Wuttke et al	416,178	NA	31152163	2019	European
T2DM	Meta-analysis	Xue et al	655,666	61,714/1178	30054458	2018	European
HDL	GLGC	Willer et al	187,167	NA	24097068	2013	Mixed
LDL	GLGC	Willer et al	173,082	NA	24097068	2013	Mixed
TC	GLGC	Willer et al	187,365	NA	24097068	2013	Mixed
TG	GLGC	Willer et al	177,861	NA	24097068	2013	Mixed
SBP	ICBP	Evangelou et al	757,601	NA	30224653	2018	European
DBP	ICBP	Evangelou et al	757,601	NA	30224653	2018	European
BMI	GIANT	Yengo et al	681,275	NA	30124842	2018	European

Abbreviations: CKD: chronic kidney disease; CKDi25: defined as the decrease of eGFR ≥ 25% of baseline accompanied by the progression from no CKD to CKD; CKD rapid3: eGFR decreases by more than 3 mL/min/1.73m^2^ per year; eGFR: estimated glomerular filtration rate; BUN: blood urea nitrogen; T2DM: type 2 diabetes; HDL: high-density lipoprotein cholesterol; LDL-C: low-density lipoprotein cholesterol; TC: total cholesterol; TG: triglyceride; SBP: systolic blood pressure; DBP: diastolic blood pressure; BMI: body mass index; GLGC: Global Lipids Genetics Consortium; ICBP: International Consortium of Blood Pressure; GIANT: Genetic Investigation of Anthropometric Traits.

### 2.2. Data source for circulating metabolome, CKD and kidney function, and 677 non-kidney diseases

About 3 million SNPs from two cohorts comprised the 452 metabolic traits across 7,824 individuals that Shin et al. evaluated to generate the summary data of single nucleotide polymorphisms (SNPs) associated with human metabolic categories [15]. The CKD summary statistics are based on a meta-analysis of the Chronic Kidney Diseases Genetics Consortium (CKDGen Consortium), which includes 23 cohorts of European descent (*n* = 480,698; 41,395 patients and 439,303 controls) [16]. The summary statistics of CKD Rapid3 (including 34,874 cases and 107,090 controls) and CKDi25 (encompassing 19,901 cases and 175,244 controls) are derived from a meta-analysis of 42 GWAS studies from the CKDGen Consortium and United Kingdom Biobank [17]. The dialysis data came from the Finngen database (r9), which contained 363,177 controls and 1004 cases [18]. The summary statistics of eGFR come from meta-analyses, including Chronic Kidney Disease Genetics (CKDGen) Consortium and UK Biobank (*n* = 1,201,909) [19]. A meta-analysis generated the BUN data, which included the summary statistics of European ancestry (*n* = 416,178) [16]. Summary statistics for 1403 disease traits were obtained from Zhou et al.’s GWAS with 408,961 white British participants and 28 million SNPs in the UK Biobank cohort [20]. The original GWAS received formal informed consent forms from each participant together with ethical approval for the program and data collection.

### 2.3. Selection of instrumental variables (IVs)

Strict screening criteria are applied to screen instrumental variables associated with blood metabolites from many perspectives in order to meet assumptions (1) (Figure 1(A)). We loosen the significance threshold of *p* < 1 × 10^−5^ in order to detect metabolite-related SNPs, considering a moderate number of metabolite-related SNPs. Next, we set up the linkage disequilibrium parameter (*r*^2^ < 0.1 and within 500 kb) to guarantee the independence of IVs. This standard has been widely used in previous studies [21,22]. The European sample from the 1000 Genomes Project served as the reference panel for linkage disequilibrium. If any relevant SNPs were not exposed in the outcome dataset, proxy SNPs were selected. Genetic instruments were looked for in proxy SNPs with high linkage disequilibrium (*r*^2^ > 0.8) at LDlink. We harmonized genetic variants by combining exposure and outcome data. In order to prevent bias in estimates of causal effects, allele mismatches must be avoided and genetic variant consistency must be guaranteed across all accessible datasets [23]. Uncertain or palindromic SNPs were not included in the analysis. In addition, in order to satisfy the minimal SNP requirements for certain MR sensitivity analyses, we eliminated less than three SNP-related metabolites [24]. We calculate the R^2^ and F statistics of each SNP to eliminate the deviation brought on by weak instruments. An F statistic greater than 10 suggests a strong instrument [25]. The calculation formulas of R^2^ and F are shown in Table S1.

### 2.4. Statistical analysis and sensitivity analysis

In UVMR analysis, inverse variance weighted (IVW) results were primarily used to assess causal relationships between blood metabolites and CKD as well as renal function. Since a summary analysis of the Wald ratios for each variant is the source of the IVW estimates [26]. The premise that all variables are valid instrument variables and that effect estimates may be biased in the presence of directional pleiotropy supports IVW’s effective statistical ability [27]. To enhance the dependability of the results, we utilized three additional methods (MR-Egger, weighted median, and Constrained Maximum Likelihood and Model Averaging (cML-MA)) to assess metabolites with significant estimates. Even though every SNP has multiple effects, MR-Egger can offer an unbiased assessment and sensitivity to outliers or inefficient values [28]. Even in cases when up to 50% of the SNP deviates from the instrument variable assumption, a reliable estimate may still be obtained using the weighted median [29]. The cML-MA method, an MR approach, combines ML with model averaging to handle both correlated and uncorrelated pleiotropic effects [30]. It differs from previous MR methods in that it does not rely on the InSIDE (Instrument Strength Independent of Direct Effect) assumption.

Sensitivity analysis is important because it focuses on heterogeneity and horizontal pleiotropy, which might significantly deviate from MR estimates. When IVs affect outcomes in ways other than those of interest, horizontal pleiotropy can be observed. Five techniques—MR pleiotropy residual sum and outlier (MR-PRESSO), Radial MR, Generalized summary data-based MR (GSMR), MR Steiger filtering, Cochran’s Q test, and MR-Egger intercept test—were used in this work to identify and adjust for heterogeneity and pleiotropy. The heterogeneity of the results is defined as *p* < .05 as determined by Cochran’s Q test [31]. To examine the directional pleiotropy and bias brought on by ineffective IVs, the MR-Egger intercept was calculated [32]. Subsequently, MR-PRESSO [33], RadialMR [34], GSMR [35], and MR Steiger filtering [36] were performed to identify outliers, and MR analysis was repeated after eliminating heterogeneous SNPs. This time, we rigorously explored the blood metabolites using several criteria that may have a causal relationship to renal function and CKD: (1) *p* value for the primary analysis was significant; (2) all four MR methods have the same direction and amplitude; and (3) the MR results showed neither horizontal pleiotropy nor heterogeneity [37].

### 2.5. Multivariable MR analysis

To ascertain that genetic variants are related to a single risk factor, MR analysis is required to prevent IVs from violating assumptions (2) and (3) (Figure 1(A)). However, in practice, several genetic variants are associated with a range of risk factors, described as pleiotropy [38]. In this situation, by combining several exposures that may interact, MVMR can correct genetic variants interaction between exposures. To put it briefly, UVMR evaluated the total effect of exposure on outcomes, whereas MVMR evaluated the direct effect of each exposure on outcomes (independent of any other exposure) [38]. In this study, for the metabolites of significant causality in UVMR analysis, MV-IVW, MVMR-Egger and MVMR-Lasso methods were used for MVMR analysis, in order to adjust the potential confounding factors, including T2DM [39], HDL [40], LDL [40], TC [40], TG [40], SBP [41], DBP [42], BMI [43]. For MVMR, we apply the MV-IVW method as the main result.

### 2.6. Phe-MR analysis for target-mediated side effects of CKD-related metabolites

We used Phe-MR analysis to investigate possible adverse effects of potential therapies that target identified metabolites to reduce the risk of CKD and improve renal function. Using 408,961 white British participants and 28 million SNPs in the UK Biobank cohort, Zhou et al.’s GWAS provided summary statistics for 1403 disease traits [20]. In order to facilitate systematic genetic analysis of certain disease traits, researchers defined disease features with ‘PheCodes’, a strategy that organizes International Classification of Disease (ICD) codes into phenotypic outcomes [20]. The disease traits in this study that had less than 500 cases were eliminated due to data availability and statistical ability. Furthermore, we select only representative phenotypes to reduce the intrinsic duplication of PheCodes and enhance the interpretability of the data. In order to better describe the targeted possible side effects of CKD-related metabolites, Phe-MR analysis was performed on a total of 677 non-renal disease traits (Table S3). The final Phe-MR results were standardized as changes in the level of metabolites, corresponding to a 10% reduction in CKD risk, based on the association between metabolites and CKD [44]. To find the side effects of focused intervention on CKD-related metabolites, we standardized Phe-MR results in this way, and we then directly compared the magnitude and direction of side effects. All MR estimates are expressed as odds ratios (OR), and the results have a 95% confidence interval (CI). Metabolites with still significant P values after confounding factors were adjusted with MVMR and entered the Phe-MR analysis. In UVMR, the observed bilateral *p* < 1.11 × 10^−4^ (Bonferroni corrected significance threshold calculated as 0.05 divided by 452 [for 452 metabolites]) was used to evaluate the statistical significance of potential causality. The statistical significance threshold of Phe-MR analysis was set to *p* < 3.69 × 10^−5^, and it was compared and corrected several times using the Bonferroni method (0.05/1354[2 identified CKD metabolites in the stage of UVMR and MVMR × 677 non-kidney diseases]). Bilateral *p* < .05 was considered as suggestive evidence of potential directional pleiotropy in the MR-Egger regression method [28]. All statistical analyses were performed with the packages of TwoSampleMR, MendelianRandomization, MRPRESSO, RadialMR, MRcML, gsmr, and ggplot2 in R (version 4.2.3; R Development Core Team).

## 3. Results

### 3.1. Strength of the instrumental variables

In this study, 452 unique blood metabolites were analyzed (Table S2). The variance of metabolites explained by genetic instruments ranged from 0.91% to 98.43%. The F statistics for the genetic instruments of blood metabolites range from 12.5834 to 1297.1255, suggesting that there is no weak instrument bias in our MR study (Tables S4–S9).

### 3.2. UVMR analysis

**Figure 2.**
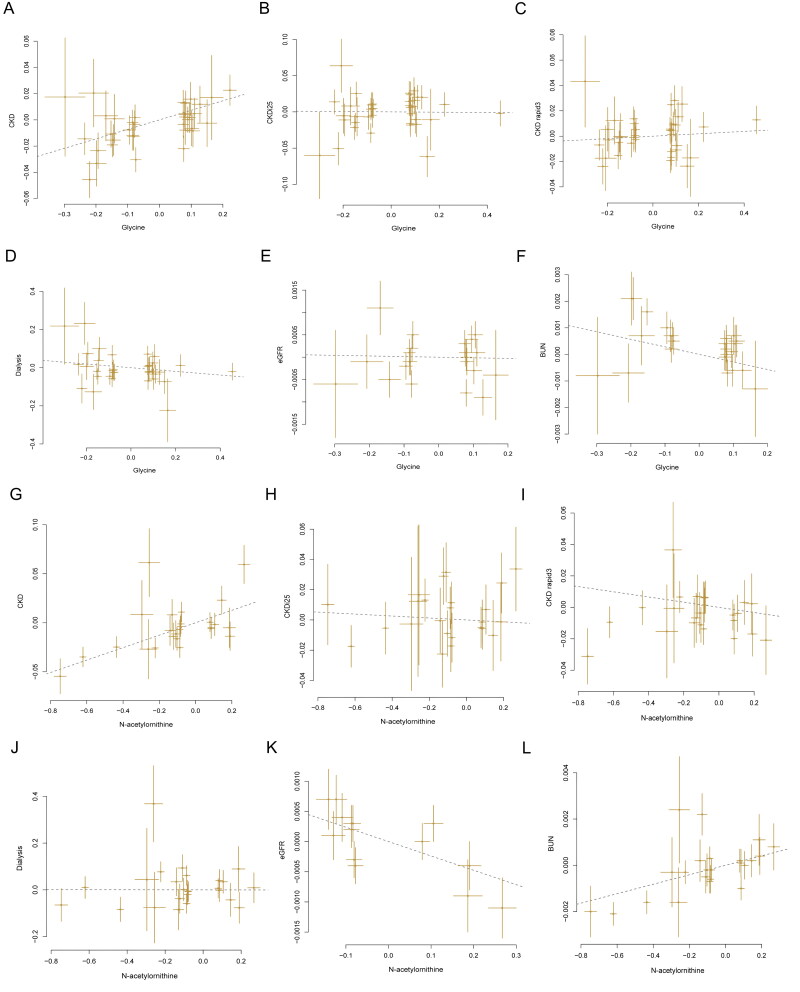
Scatter plot of SNPs associated with circulating metabolites having etiologic associations with CKD and renal function. Abbreviations: CKD: chronic kidney disease; CKDi25: defined as the decrease of eGFR ≥ 25% of baseline accompanied by the progression from no CKD to CKD; CKD rapid3: eGFR decreases by more than 3 mL/min/1.73m^2^ per year; eGFR: estimated glomerular filtration rate; BUN: blood urea nitrogen.

In primary MR analysis, the IVW method was used to estimate the causal relationship between 452 blood metabolites and CKD as well as renal function, as shown in Figure 2, Table 2 and 3 and Tables S4–S9. The MR results showed that each 1-SD higher mannose (OR 1.9367; 95% CI 1.5026, 2.4961; *p* = 3.30 × 10^−7^); N-acetylornithine (OR 1.2529; 95% CI 1.1539,1.3603; *p* = 7.84 × 10^−8^) and glycine (OR 1.6628; 95% CI 1.3867,1.9938; *p* = 4.03 × 10^−8^) were associated with an increased risk of CKD; the MR results showed that higher X-11204 (Beta 0.0262; 95% CI 0.0138, 0.0385; *p* = 3.45 × 10^−5^); higher X-11529 (Beta 0.0046; 95% CI 0.0030, 0.0061; *p* = 5.50 × 10^−9^) and X-11538 (Beta 0.0097; 95% CI 0.0061,0.0133; *p* = 1.34 × 10^−7^) were associated with higher eGFR, however, higher 5-dodecenoate (12:1n7) (Beta −0.0124; 95% CI −0.0182, −0.0066; *p* = 2.47 × 10^−5^) was associated with lower eGFR; the MR results showed that higher N-acetylornithine (Beta 0.0122; 95% CI 0.0079,0.0164; *p* = 1.47 × 10^−8^) was associated with higher BUN, however, higher tryptophan betaine (Beta −0.0100; 95% CI −0.0148, −0.0051; *p* = 5.35 × 10^−5^) was associated with lower BUN (Figure 2, Tables 2 and 3 and Tables S4–S9). Subsequently, we conducted a series of sensitivity analyses to assess the robustness we found in the primary analysis. As shown in Tables 2 and 3, Tables S4–S9 and Figure S1, there are still similar results in a sensitivity analysis using MR-Egger, weighted median, and cML-MA, MR-Egger regression showed no evidence of pleiotropy and Cochran’s Q test did not show evidence of heterogeneity. The reverse MR analyses revealed no significant causal effect of genetic predisposition to any CKD and renal function on the risk of serum metabolite (Tables S10–S15).

**Table 2.** MR analyses for circulating metabolites having etiologic associations with CKD and renal function.

Exposure	Outcome	Method	OR/beta (95% CI)	*p* Value
Glycine, unadjusted	CKD	IVW	1.6628 (1.3867, 1.9938)	4.03E − 08
Glycine, unadjusted	CKD	MR-PRESSO	1.7292 (1.4317, 2.0885)	3.01E − 06
Glycine, adjusted	CKD	MV-IVW	1.1437 (1.0286, 1.2716)	1.31E − 02
Glycine, adjusted	CKD	MVMR-Lasso	1.1035 (1.0047, 1.2120)	3.95E − 02
N-acetylornithine, unadjusted	CKD	IVW	1.2529 (1.1539, 1.3603)	7.84E − 08
N-acetylornithine, unadjusted	CKD	MR-PRESSO	1.2680 (1.1617, 1.3842)	2.48E − 05
N-acetylornithine, adjusted	CKD	MV-IVW	1.1229 (1.0601, 1.1893)	7.83E − 05
N-acetylornithine, adjusted	CKD	MVMR-Lasso	1.1288 (1.0735, 1.1870)	2.30E − 06
Glycine, unadjusted	CKDi25	IVW	1.1466 (0.8688, 1.5133)	3.34E − 01
Glycine, unadjusted	CKDi25	MR-PRESSO	1.2127 (0.8971, 1.6393)	2.19E − 01
Glycine, adjusted	CKDi25	MV-IVW	1.0136 (0.8885, 1.1562)	8.41E − 01
Glycine, adjusted	CKDi25	MVMR-Lasso	1.0136 (0.8885, 1.1562)	8.41E − 01
N-acetylornithine, unadjusted	CKDi25	IVW	1.0412 (0.9271, 1.1693)	4.96E − 01
N-acetylornithine, unadjusted	CKDi25	MR-PRESSO	1.0514 (0.9464, 1.1679)	3.61E − 01
N-acetylornithine, adjusted	CKDi25	MV-IVW	1.0719 (0.9982, 1.1510)	5.61E − 02
N-acetylornithine, adjusted	CKDi25	MVMR-Lasso	1.0719 (0.9982, 1.1510)	5.61E − 02
Glycine, unadjusted	CKD rapid3	IVW	1.1632 (0.9710, 1.3933)	1.01E − 01
Glycine, unadjusted	CKD rapid3	MR-PRESSO	1.1972 (0.9816, 1.4600)	8.54E − 02
Glycine, adjusted	CKD rapid3	MV-IVW	1.1348 (1.0347, 1.2446)	7.30E − 03
Glycine, adjusted	CKD rapid3	MVMR-Lasso	1.1338 (1.0355, 1.2415)	6.65E − 03
N-acetylornithine, unadjusted	CKD rapid3	IVW	1.0381 (0.9581, 1.1247)	3.61E − 01
N-acetylornithine, unadjusted	CKD rapid3	MR-PRESSO	1.0306 (0.9593, 1.1071)	4.19E − 01
N-acetylornithine, adjusted	CKD rapid3	MV-IVW	1.0653 (1.0135, 1.1199)	1.30E − 02
N-acetylornithine, adjusted	CKD rapid3	MVMR-Lasso	1.0635 (1.0126, 1.1170)	1.39E − 02
Glycine, unadjusted	Dialysis	IVW	0.8090 (0.3472, 1.8849)	6.23E − 01
Glycine, unadjusted	Dialysis	MR-PRESSO	0.8090 (0.3990, 1.6402)	5.61E − 01
Glycine, adjusted	Dialysis	MV-IVW	0.8885 (0.5629, 1.4026)	6.12E − 01
Glycine, adjusted	Dialysis	MVMR-Lasso	0.9216 (0.5890, 1.4419)	7.21E − 01
N-acetylornithine, unadjusted	Dialysis	IVW	1.1109 (0.7411, 1.6652)	6.11E − 01
N-acetylornithine, unadjusted	Dialysis	MR-PRESSO	0.9532 (0.6410, 1.4174)	8.15E − 01
N-acetylornithine, adjusted	Dialysis	MV-IVW	1.0950 (0.8567, 1.3997)	4.69E − 01
N-acetylornithine, adjusted	Dialysis	MVMR-Lasso	1.0744 (0.8451, 1.3659)	5.58E − 01
Glycine, unadjusted	eGFR	IVW	0.0065 (−0.0034, 0.0163)	2.01E − 01
Glycine, unadjusted	eGFR	MR-PRESSO	0.0043 (−0.0049, 0.0135)	3.67E − 01
Glycine, adjusted	eGFR	MV-IVW	−0.0055 (−0.0100, −0.0010)	1.76E − 02
Glycine, adjusted	eGFR	MVMR-Lasso	−0.0038 (−0.0069, −0.0007)	1.58E − 02
N-acetylornithine, unadjusted	eGFR	IVW	−0.0116 (−0.0183, −0.0049)	6.83E − 04
N-acetylornithine, unadjusted	eGFR	MR-PRESSO	−0.0116 (−0.0165, −0.0066)	8.07E − 04
N-acetylornithine, adjusted	eGFR	MV-IVW	−0.0071 (−0.0098, −0.0043)	5.15E − 07
N-acetylornithine, adjusted	eGFR	MVMR-Lasso	−0.0054 (−0.0073, −0.0035)	3.11E − 08
Glycine, unadjusted	BUN	IVW	−0.0065 (−0.0235, 0.0104)	4.50E − 01
Glycine, unadjusted	BUN	MR-PRESSO	−0.0148 (−0.0319, 0.0023)	1.04E − 01
Glycine, adjusted	BUN	MV-IVW	−0.0087 (−0.0160, −0.0015)	1.83E − 02
Glycine, adjusted	BUN	MVMR-Lasso	−0.0054 (−0.0108, 0.0001)	5.01E − 02
N-acetylornithine, unadjusted	BUN	IVW	0.0122 (0.0079, 0.0164)	1.47E − 08
N-acetylornithine, unadjusted	BUN	MR-PRESSO	0.0112 (0.0068, 0.0156)	6.71E − 05
N-acetylornithine, adjusted	BUN	MV-IVW	0.0084 (0.0045, 0.0122)	2.30E − 05
N-acetylornithine, adjusted	BUN	MVMR-Lasso	0.0071 (0.0041, 0.0102)	4.58E − 06

Abbreviations: CKD: chronic kidney disease; CKDi25: defined as the decrease of eGFR ≥ 25% of baseline accompanied by the progression from no CKD to CKD; CKD rapid3: eGFR decreases by more than 3 mL/min/1.73m^2^ per year; eGFR: estimated glomerular filtration rate; BUN: blood urea nitrogen; OR: odds ratio; CI: confidence interval; IVW: inverse variance weighted; MV-IVW: multivariable inverse variance weighted; unadjusted: represent the univariable Mendelian randomization (UVMR) results; adjusted: represent the multivariable Mendelian randomization (MVMR) results after adjusting the potential confounding factors, including T2DM, HDL, LDL, TC, TG, SBP, DBP, BMI.

**Table 3.** GSMR analyses for circulating metabolites having etiologic associations with CKD and renal function.

Exposure	Outcome	Method	OR/beta (95% CI)	*p* Value
Glycine	CKD	GSMR	1.0752 (1.0379, 1.1139)	5.66E − 05
Glycine	CKDi25	GSMR	0.9977 (0.9464, 1.0519)	9.32E − 01
Glycine	CKD rapid3	GSMR	1.0095 (0.9734, 1.0470)	6.10E − 01
Glycine	Dialysis	GSMR	0.9084 (0.7719, 1.0692)	2.48E − 01
Glycine	eGFR	GSMR	−0.0001 (−0.0017,0.0014)	8.63E − 01
Glycine	BUN	GSMR	−0.0028 (−0.0054, −0.0002)	3.19E − 02
N-acetylornithine	CKD	GSMR	1.0655 (1.0322, 1.0999)	8.93E − 05
N-acetylornithine	CKDi25	GSMR	0.9936 (0.9474, 1.0420)	7.90E − 01
N-acetylornithine	CKD rapid3	GSMR	0.9836 (0.9530, 1.0153)	3.07E − 01
N-acetylornithine	Dialysis	GSMR	0.9988 (0.8649, 1.1534)	9.87E − 01
N-acetylornithine	eGFR	GSMR	−0.0024 (−0.0041, −0.0006)	8.20E − 03
N-acetylornithine	BUN	GSMR	0.0020 (0.0002, 0.0039)	3.05E − 02

Abbreviations: CKD: chronic kidney disease; CKDi25: defined as the decrease of eGFR ≥ 25% of baseline accompanied by the progression from no CKD to CKD; CKD rapid3: eGFR decreases by more than 3 mL/min/1.73m^2^ per year; eGFR: estimated glomerular filtration rate; BUN: blood urea nitrogen; OR: odds ratio; CI: confidence interval; GSMR: generalized summary data-based Mendelian randomization.

### 3.3. MVMR analysis

MVMR analysis was performed to evaluate the direct effects of 8 metabolites screened by UVMR (5-decanoate (12:1n7), glycine, mannose, N-acetylornithine, tryptophan betaine, X-11204, X-11529, X-11538) on CKD and renal function, and to adjust potential risk factors, including T2DM, HDL, LDL, TC, TG, SBP, DBP, BMI. The MVMR results showed that higher glycine (OR 1.1437, 95% CI 1.0286, 1.2716, *p* = 1.31 × 10^−2^); N-acetylornithine (OR 1.1229; 95% CI 1.0601, 1.1893, *p* = 7.83 × 10^−5^); X-11204 (OR 1.1706; 95% CI 1.0053,1.3631, *p* = 4.26 × 10^−2^); and X-12206 (OR 1.1229; 95% CI 1.0068, 1.2523, *p* = 3.74 × 10^−2^) were associated with an increased risk of CKD after adjusting for those potential risk factors (Table S16); MVMR results showed that higher Glycine (OR 1.1348, 95% CI 1.0347, 1.2446, *p* = 7.30 × 10^−3^) and N-acetylornithine (OR 1.0653; 95% CI 1.0135, 1.1199, *p* = 1.30 × 10^−2^) were associated with an increased risk of CKD rapid3 after adjusting for those potential risk factors (Table S18); MVMR results showed that higher glycine (Beta −0.0055, 95% CI −0.0100, −0.0010, *p* = 1.76 × 10^−2^), N-acetylornithine (Beta −0.0071; 95% CI −0.0098, −0.0043, *p* = 5.15 × 10^−7^) and X-12206 (Beta −0.0047; 95% CI −0.0082, −0.0012, *p* = 7.93 × 10^−3^) were associated with lower eGFR after adjusting for those potential risk factors (Table S20), however, X-11529 (Beta 0.0017; 95% CI 0.0002, 0.0032; *p* = 2.54 × 10^−2^) has a significant positive correlation with eGFR after adjusting for those potential risk factors (Table S20). MVMR results showed that higher glycine (Beta −0.0087, 95% CI −0.0160, −0.0015, *p* = 1.83 × 10^−2^) was associated with lower BUN after adjusting for those potential risk factors (Table S21), however, higher N-acetylornithine (Beta 0.0084; 95% CI 0.0045, 0.0122, *p* = 2.30 × 10^−5^) was associated with higher BUN after adjusting for those potential risk factors (Table S21). When adjusting for confounders to observe their causal relationship with the risk of CKD alone, X-11204 had pleiotropic effects (Table S16), which may be due to the influence of other potential confounders. In addition, our MVMR results showed that no causal relationship between metabolites and CKDi25 and Dialysis was found (Table S17 and Table S19). When other metabolites were analyzed by MVMR, MR-Egger regression did not show evidence of pleiotropic effects (Tables S16–S21). All in all, after adjusting for these confounding factors (T2DM, HDL, LDL, TC, TG, SBP, DBP, BMI), there is still a significant causal relationship between Glycine, N-acetylornithine and CKD, CKD rapid3, eGFR, and BUN (Table 2 and Table S22).

### 3.4. Phe-MR analysis for the associations between identified metabolites and 677 non-kidney diseases

To investigate the possible adverse effects of the identified metabolites, additional Phe-MR analysis was carried out to thoroughly evaluate their impact on the risk of 677 non-kidney diseases. Phe-MR, which targets a specific metabolite and reduces the risk of CKD by 10%, gives standardized results, unlike previous MR. Thus, if each metabolite is used to prevent CKD, the associations that result can be interpreted as the expected side effects. Three relationships in total reached the Bonferroni-corrected significance threshold of *p* < 3.69 × 10^−5^ (0.05/1354 [2 metabolites × 677 diseases]) in the Phe-MR study conducted using the IVW method. One disease relationship for N-acetylornithine and two significant disease associations for glycine were discovered in the sensitivity analyses using the MR-Egger, weighted median, and cML-MA methods. In summary, there are three noticeable correlations between various non-renal diseases and targeting glycine and N-acetylornithine (Table 4, Tables S23 and S24). In summary, lowering circulating glycine has an adverse effect on the risk of two diseases (cholecystitis and cholelithiasis), whereas decreasing serum N-acetylornithine has a negative impact on tinnitus. The most significant diseases associated with glycine and N-acetylornithine were cholelithiasis and cholecystitis (OR per 10% reduction in CKD risk, 1.1097; 95% CI, 1.0589–1.1630; *p* = 1.33 × 10^−5^) and tinnitus (OR per 10% reduction in CKD risk, 1.7369; 95% CI, 1.3445–2.2438; *p* = 2.38 × 10^−5^) (Table 4).

**Table 4.** Phe-MR analyses for causal associations of glycine and N-acetylornithine with the risk of multiple non-kidney diseases.

			IVW		MR-Egger regression
Exposure	PheCode	Outcome	OR (95% CI)	*p* Value	*p* Value
Glycine	574	Cholelithiasis and cholecystitis	1.1097 (1.0589, 1.1630)	1.33E − 05	6.79E − 01
Glycine	574.1	Cholelithiasis	1.1087 (1.0562, 1.1638)	3.02E − 05	5.58E − 01
N-acetylornithine	389.4	Tinnitus	1.7369 (1.3445, 2.2438)	2.38E − 05	4.01E − 01

Abbreviations: IVW: inverse variance weighted; OR: odds ratio; CI: confidence interval.

## 4. Discussion

This is the first systematic MR study to completely evaluate the potential for targeted clinical side effects of possible CKD mediators in human blood metabolic groups. Among the 452 blood metabolites, we mainly identified N-acetylornithine and glycine as possible causal mediators for CKD. Specifically, CKD, CKD rapid3, eGFR, and BUN were significantly impacted by glycine and N-acetylornithine even after these risk factors (T2DM, HDL, LDL, TC, TG, SBP, DBP, and BMI) were adjusted by MVMR. In order to investigate the targeted side effects associated with possible CKD therapy through intervention with this discovered metabolite, we further performed a Phe-MR study. Reducing the serum glycine level in Phe-MR analysis exhibited adverse effects on cholecystitis and cholelithiasis. The risk of tinnitus is negatively impacted by lowering the level of serum N-acetylornithine.

The smallest amino acid and the one without D or L enantiomers is glycine. Humans obtain glycine from two sources: diet and endogenous synthesis, which occurs in the liver and kidneys. Choline, glycine oxalate, betaine (trimethylglycine), glucose (*via* serine), during the endogenous manufacture of L-carnitine, and likely from threonine can all be converted into glycine [45]. Meanwhile, the heme group, purines, creatine, collagen, and glutathione synthesis all depend on glycine. Patients with advanced CKD often develop uremia due to the accumulation of uremic toxins, which can lead to several adverse medical outcomes. These include progressive CKD accompanied by pathophysiologic processes such as renal fibrosis, atherosclerosis, and an increased incidence of cardiovascular events. Oxidative stress plays a significant role in inducing damage to cellular proteins, lipids, and DNA, while also exacerbating inflammation, further contributing to kidney dysfunction [46]. Additionally, kidney fibrosis is characterized by the excessive deposition of extracellular matrix components, particularly collagen, within the renal parenchyma [46]. Collagen synthesis in patients with organ fibrosis produces a unique molecular structure where approximately one-third of the amino acids are glycine [47]. Glycine serves as a critical substrate for collagen production; however, excessive glycinergic signaling has been implicated in promoting renal fibrosis progression. Investigating the interplay between oxidative stress, inflammation, and fibrosis represents an important area for future research to better understand disease mechanisms and develop targeted therapeutic interventions. We performed Phe-MR analysis to explore the adverse effects of targeting glycine metabolites. The findings demonstrated that cholelithiasis and cholecystitis were adversely affected when serum glycine levels were decreased. As a result, serum glycine may be an acceptable therapeutic target for preventing the progress of CKD and the decline of renal function; nevertheless, potential negative side effects need to be carefully considered in the clinic.

Protein acetylation ability variation has long been known to have pathogenic significance in a variety of diseases [48]. Acetylation is one of the most frequent modifications to post-translational protein modification that takes place in the context of chronic diseases [49]. For instance, renal damage brought on by drugs and toxins has also been related to acetylation abnormalities [50]. Histone deacetylase 3 (HDAC3) inhibition causes PPAR-γ to bind to cotranscription activators with increased acetylations of PPAR-γ K240 and K265. This increases the transcriptional capacity of PPAR-γ toward Klotho and other downstream renoprotective genes, consequentially leading to the renoprotections [51]. Previous studies have shown that higher circulating levels of N-acetylornithine were associated with CKD and renal function [52,53], the driver of this relationship can be explained by the common missense SNP rs13538 in the NAT8 (N-acetyltransferase 8) gene. Furthermore, eGFR, cystatin C, and serum creatinine all had a strong correlation with N-acetylornithine, according to metabonomic analysis [54]. Additionally, our Phe-MR analysis adds to the understanding of possible negative impacts associated with lowering N-acetylornithine in order to prevent CKD. According to Phe-MR studies, the risk of tinnitus increases when serum N-acetylornithine levels are lowered. Ultimately, even though N-acetylornithine might be a therapeutic target for improving renal function and CKD, it is best undertaken after carefully considering the benefits and drawbacks of the medical treatment.

Excessive protein consumption may adversely affect kidney function through several mechanisms. First, high protein intake can lead to vasodilation of renal arterioles, resulting in glomerular hypertension and increased ultrafiltration rates. These changes may exacerbate the progression of preexisting CKD [55]. Second, elevated consumption of red and processed meats is associated with increased blood pressure due to high sodium chloride intake, as well as metabolic acidosis, mitochondrial oxidative stress triggered by saturated fats, DNA damage caused by N-nitroso compounds, and enhanced accumulation of protein catabolite end-products, such as p-cresyl sulfate, indoxyl sulfate, and trimethylamine oxide [56,57]. As a result, nephrologists recommend that CKD patients restrict their protein intake to minimize the accumulation of these harmful molecules and reduce the risk of CKD progression and uremic symptoms [56]. A low-protein diet (LPD) has long been recommended for managing CKD due to its potential protective effects on renal hemodynamics. Additionally, limiting protein intake from animal sources and shifting toward plant-based protein alternatives is associated with favorable outcomes, such as reduced levels of uremic toxins, correction of metabolic acidosis, decreased phosphorus load, and improved control of metabolic bone disorders. Based on our findings, CKD patients are advised to reduce their consumption of foods containing glycine and N-acetylornithine to mitigate the nephrotoxic effects of protein overload and delay CKD progression.

Numerous significant clinical and public health implications emerge from our findings. In clinical settings, CKD typically goes unnoticed until it progresses to a later stage. The optimum method to detect and treat CKD early has yet to be agreed upon, despite the availability of a number of simple and inexpensive diagnostic tests [58,59]. Given that the clinical signs and symptoms of CKD are often imperceptible and silent [60], it is crucial to discover some potential biomarkers to detect high-risk individuals with CKD early. Based on our findings, glycine, and N-acetylornithine may be applied as therapeutic targets to prevent CKD and improve renal function. They may also be potential biomarkers of renal function decline and CKD. Obviously, more clinical trials are required to verify the safety and efficacy of glycine and N-acetylornithine in preventing CKD and improving renal function. However, the verified results will contribute to accurate CKD prevention.

There are various benefits to the current research. First, this systematic MR analysis offers novel insights into the pathogenic mediators of renal function and CKD based on genomic and metabonomic data. Second, in order to assess the causal independence of metabolites screened by UVMR on CKD and renal function, confounding factors were corrected using MVMR. To be able to completely evaluate the side effects of interventions against the identified metabolites, we lastly performed a Phe-MR analysis. This may further aid in the prioritization of therapeutic targets in drug development and clinical trials. However, our research also has some limitations. First, only 452 different metabolites were included in our MR study, which represents a very small percentage of blood metabolites. Thus, more research is required to determine the relationship between blood metabolites and renal function and CKD. Second, only individuals of European descent were taken into account in this study, which reduced the bias caused by demographic stratification but constrained the interethnic findings we could reach. To validate our results, further research on non-Europeans is required [61]. Third, patients in the PheCode system are diagnosed in the hospital, so the disease characteristics of low admission rates may not be well represented.

## 5. Conclusion

The probability of renal function decline and the occurrence of CKD may currently be causally mediated by glycine and N-acetylornithine, according to systematic MR analysis. Information on the priority of pharmacological targets to prevent the progression of CKD and renal function can be obtained from the side effect description. Glycine and N-acetylornithine may be promising drug targets for the prevention of CKD and renal function decline.

## Supplementary Material

LRNF-2024-CS-1772.R2_figure.zip

Supplementray_Tables.xlsx

## Data Availability

The original contributions presented in the study are included in the article, further inquiries can be directed to the corresponding authors.
