# Supplementary figures and images for: Causal associations between circulating metabolites and chronic kidney disease: a Mendelian randomization study
Source: Ren Fail. 2025 Apr 29;47(1):2498090. doi: 10.1080/0886022X.2025.2498090 (PMC12044913; doi:10.1080/0886022X.2025.2498090)

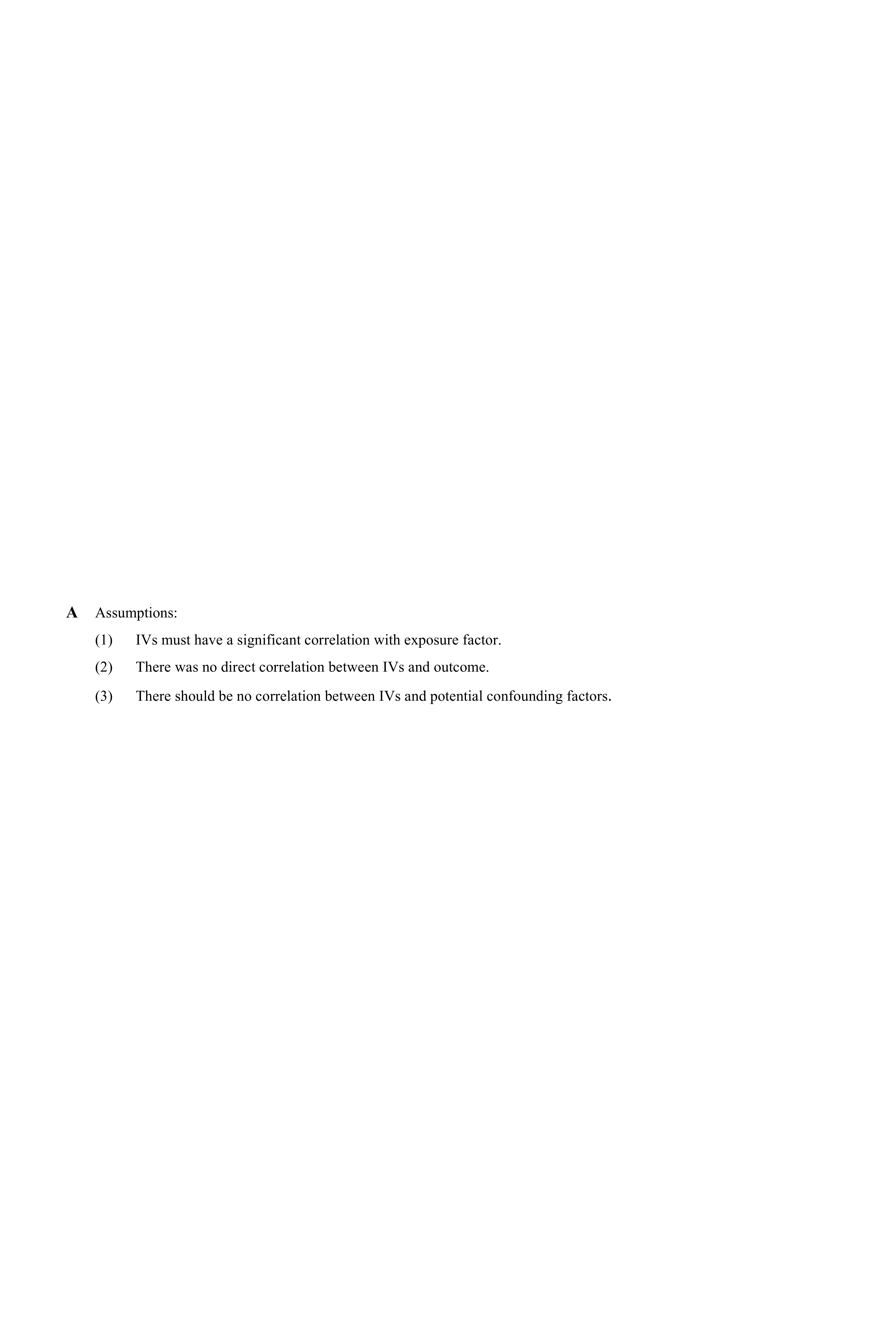

Supplement: LRNF-2024-CS-1772.R2_figure.zip [file IRNF_A_2498090_SM3483.zip › 1A.TIF]

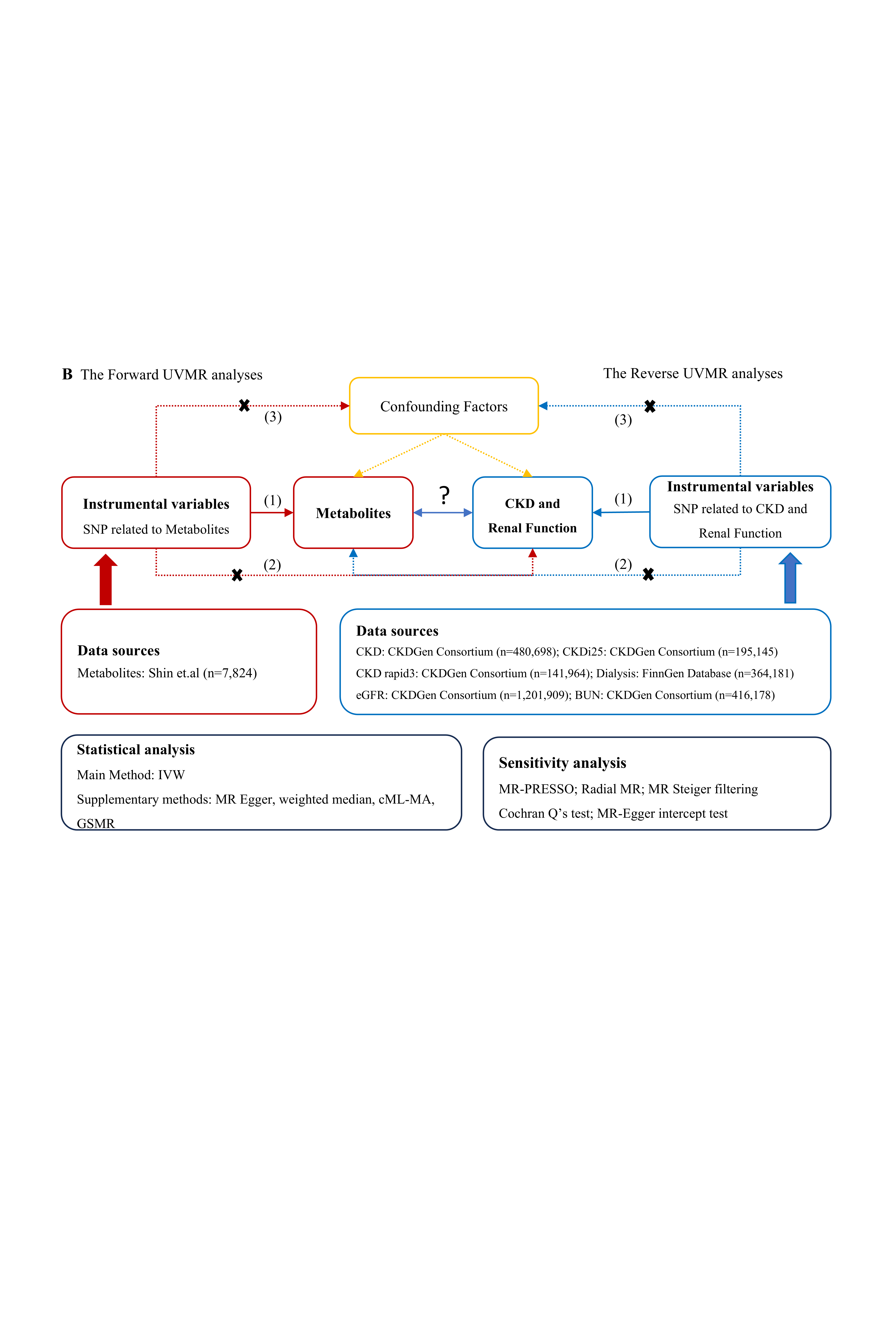

Supplement: LRNF-2024-CS-1772.R2_figure.zip [file IRNF_A_2498090_SM3483.zip › 1B.TIF]

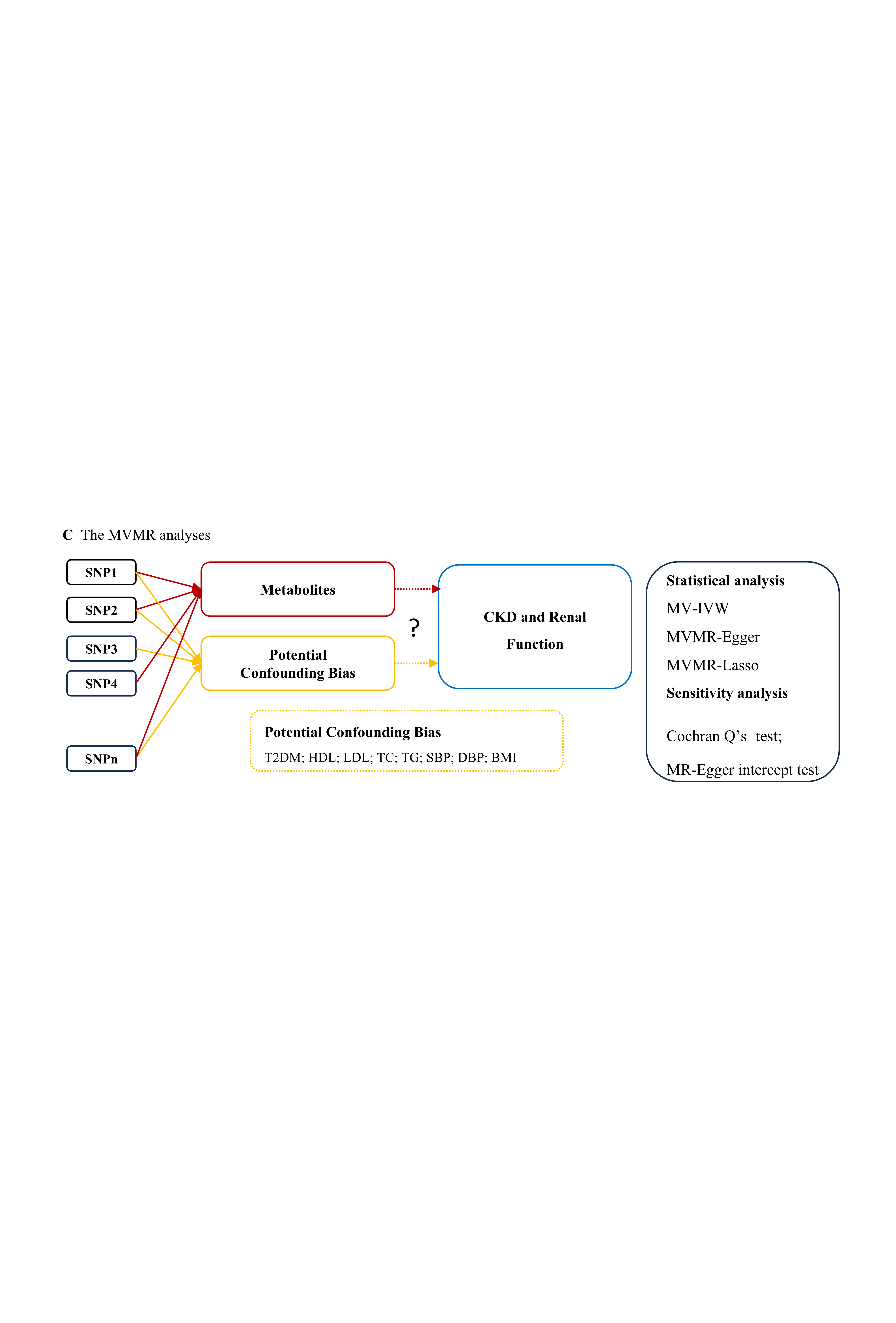

Supplement: LRNF-2024-CS-1772.R2_figure.zip [file IRNF_A_2498090_SM3483.zip › 1C.TIF]

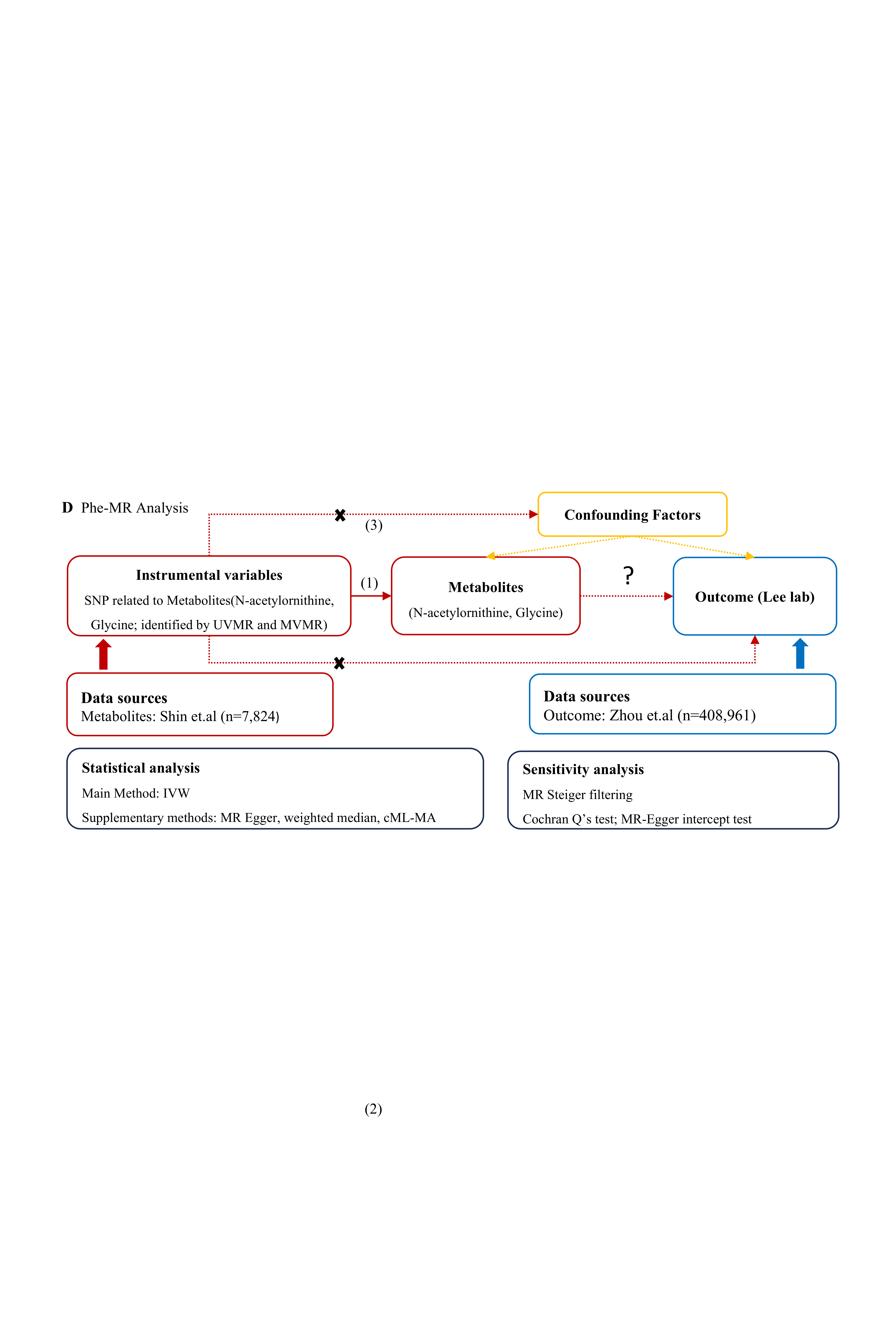

Supplement: LRNF-2024-CS-1772.R2_figure.zip [file IRNF_A_2498090_SM3483.zip › 1D.TIF]

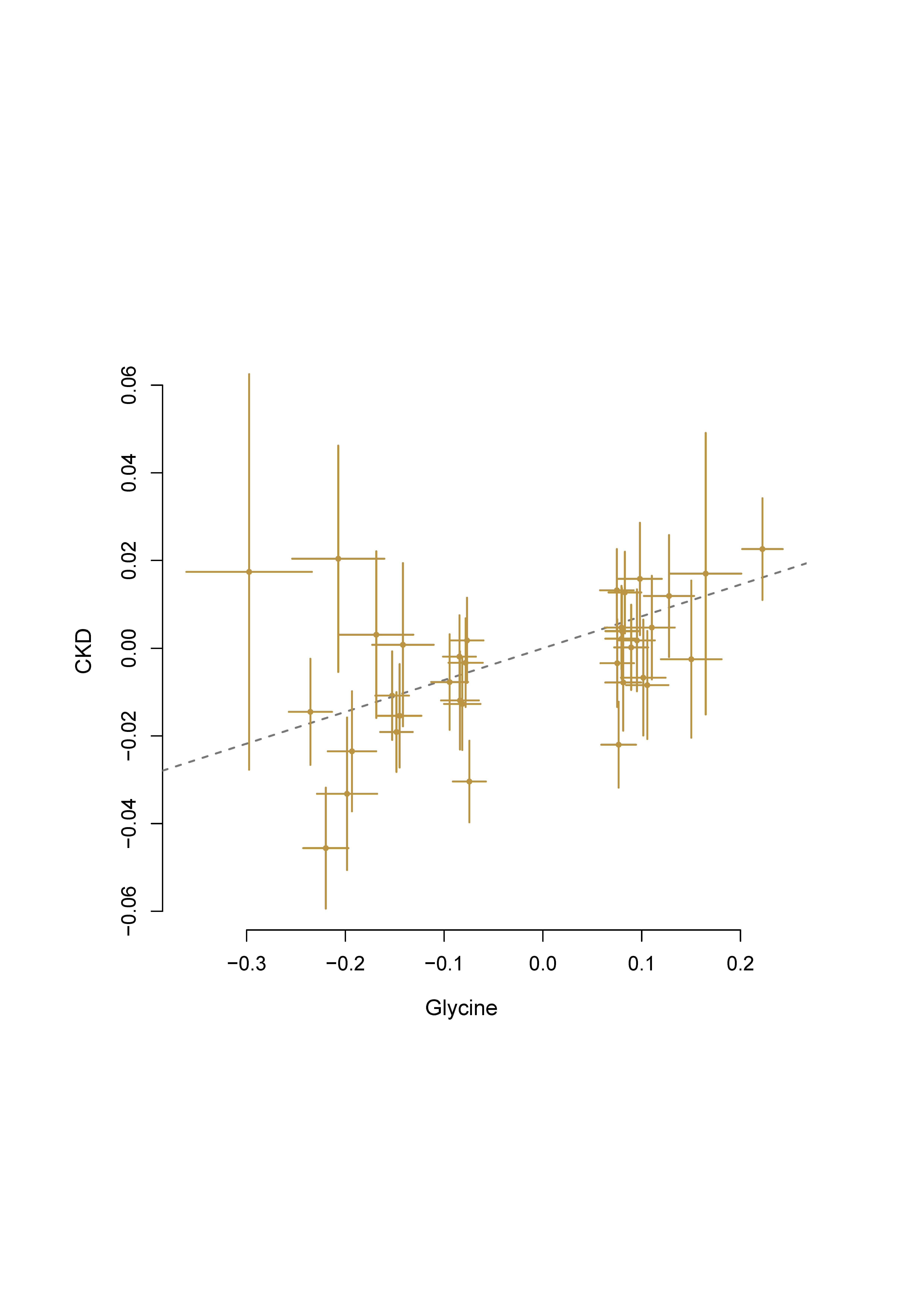

Supplement: LRNF-2024-CS-1772.R2_figure.zip [file IRNF_A_2498090_SM3483.zip › 2A.tif]

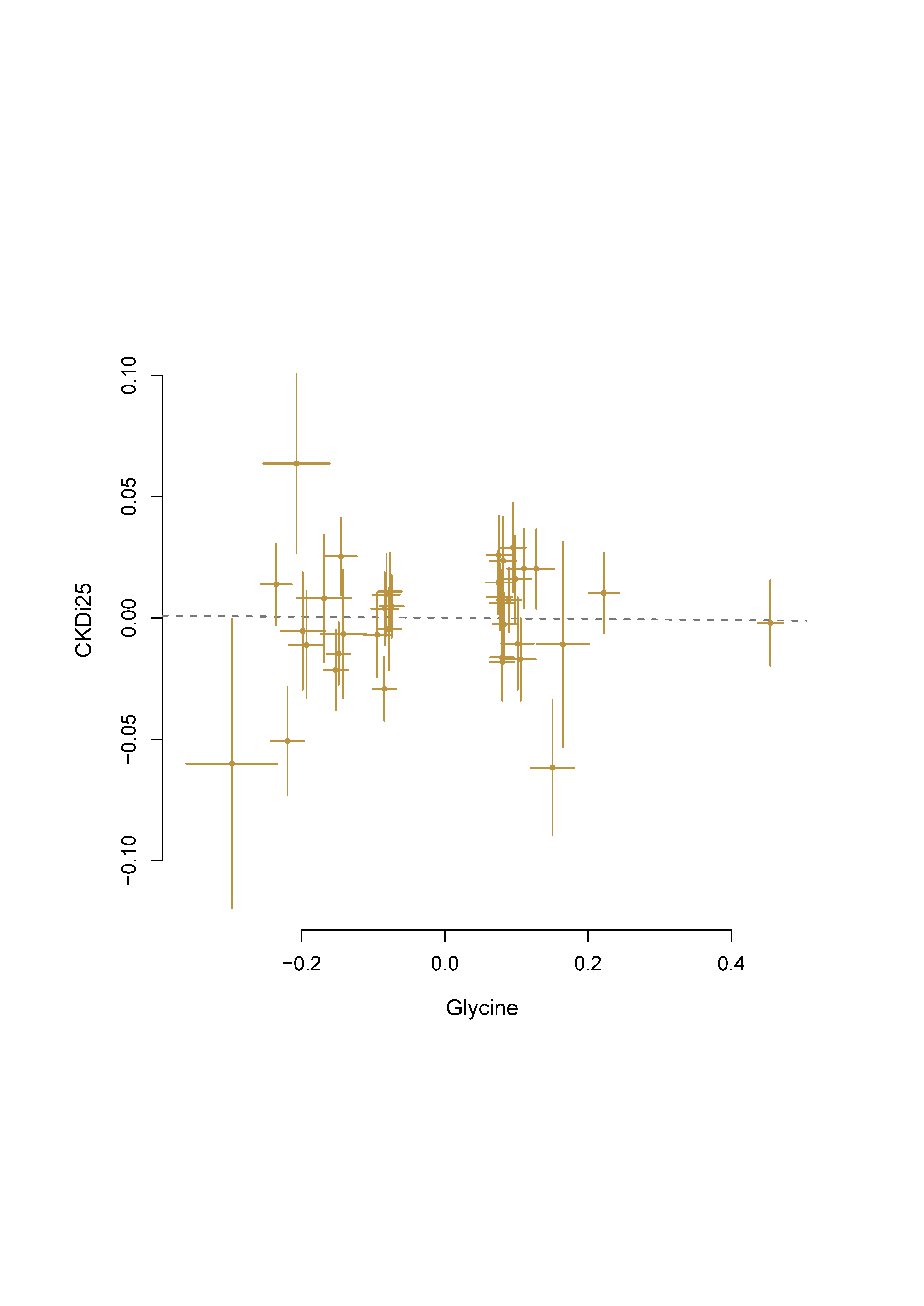

Supplement: LRNF-2024-CS-1772.R2_figure.zip [file IRNF_A_2498090_SM3483.zip › 2B.tif]

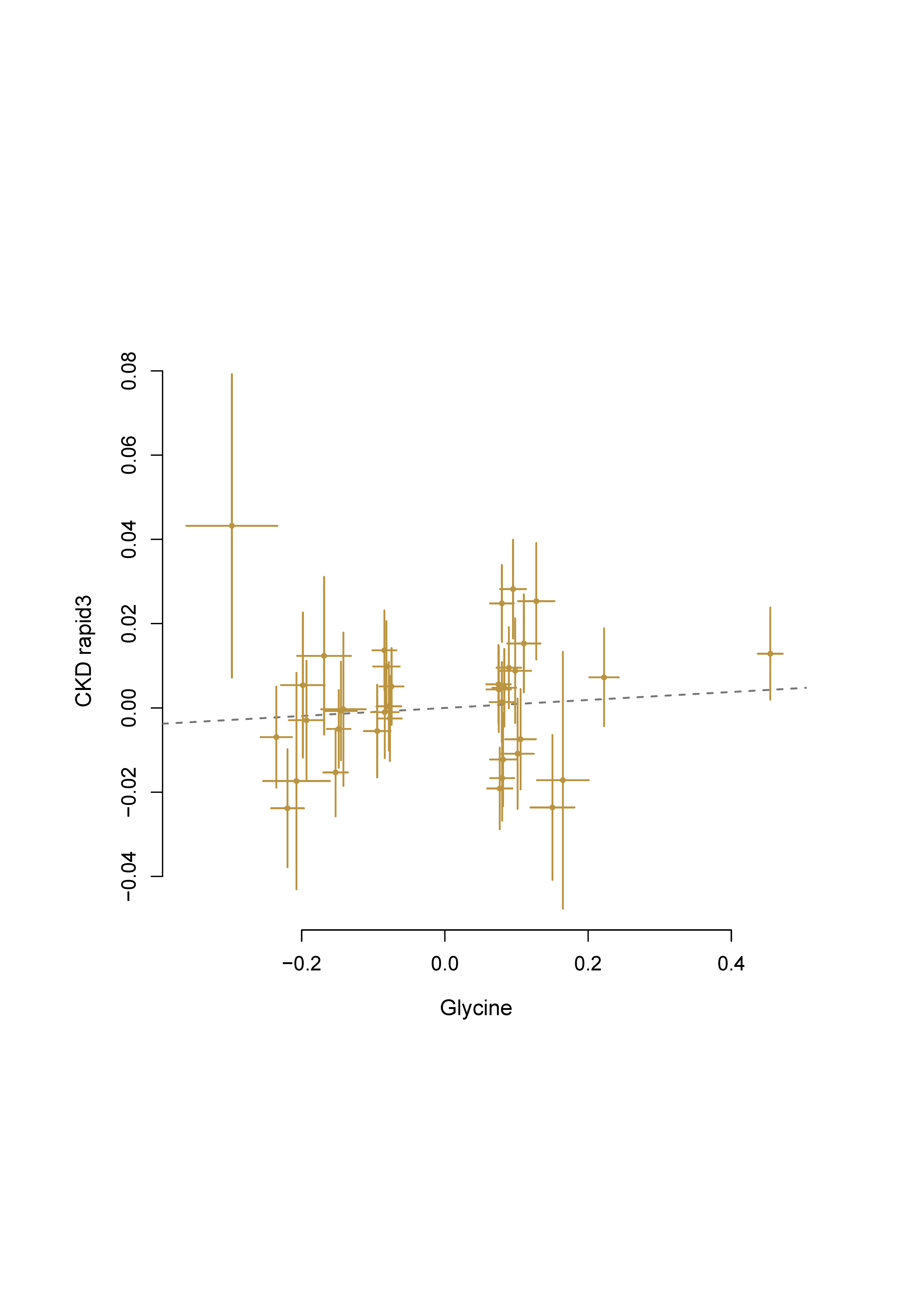

Supplement: LRNF-2024-CS-1772.R2_figure.zip [file IRNF_A_2498090_SM3483.zip › 2C.tif]

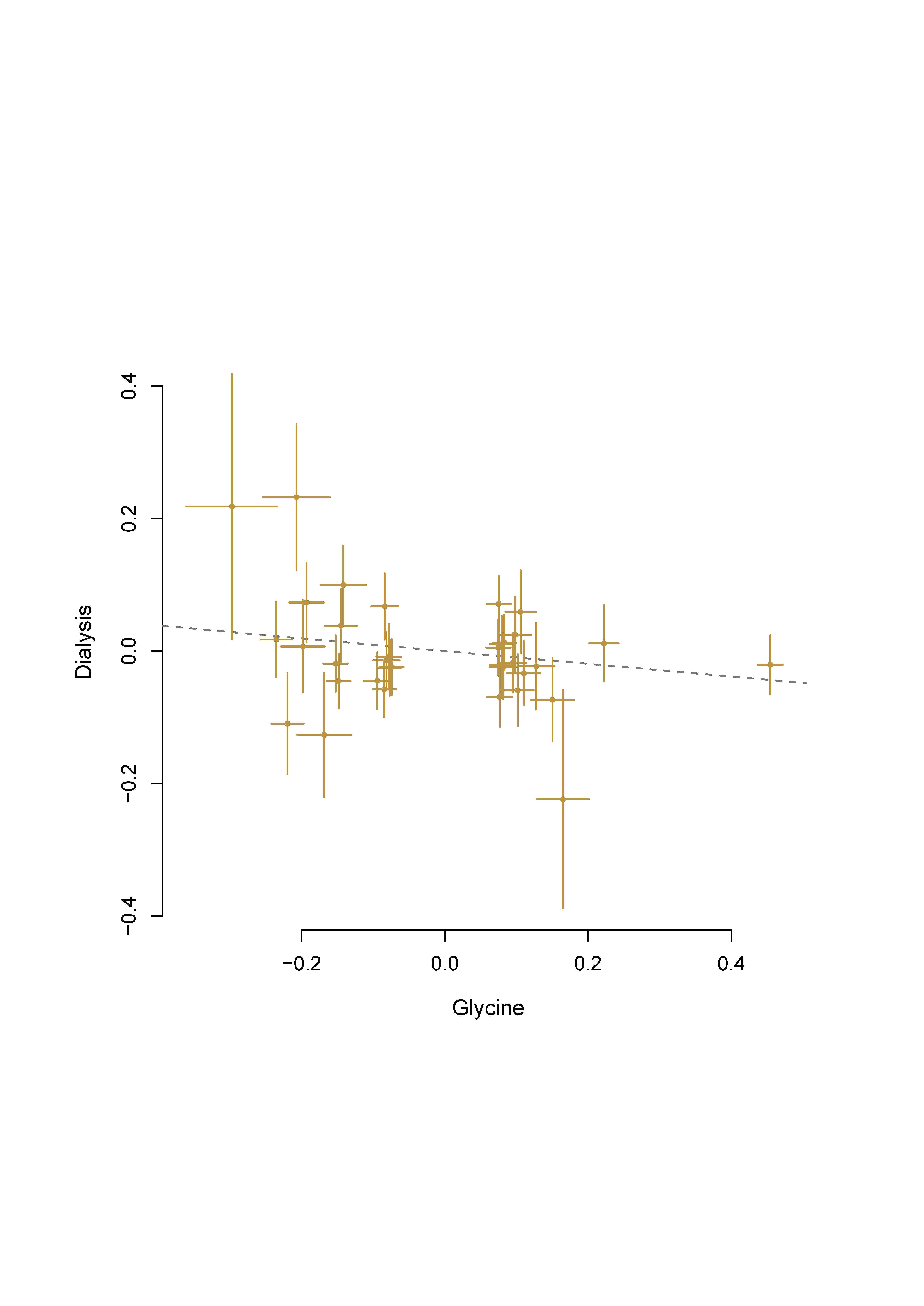

Supplement: LRNF-2024-CS-1772.R2_figure.zip [file IRNF_A_2498090_SM3483.zip › 2D.tif]

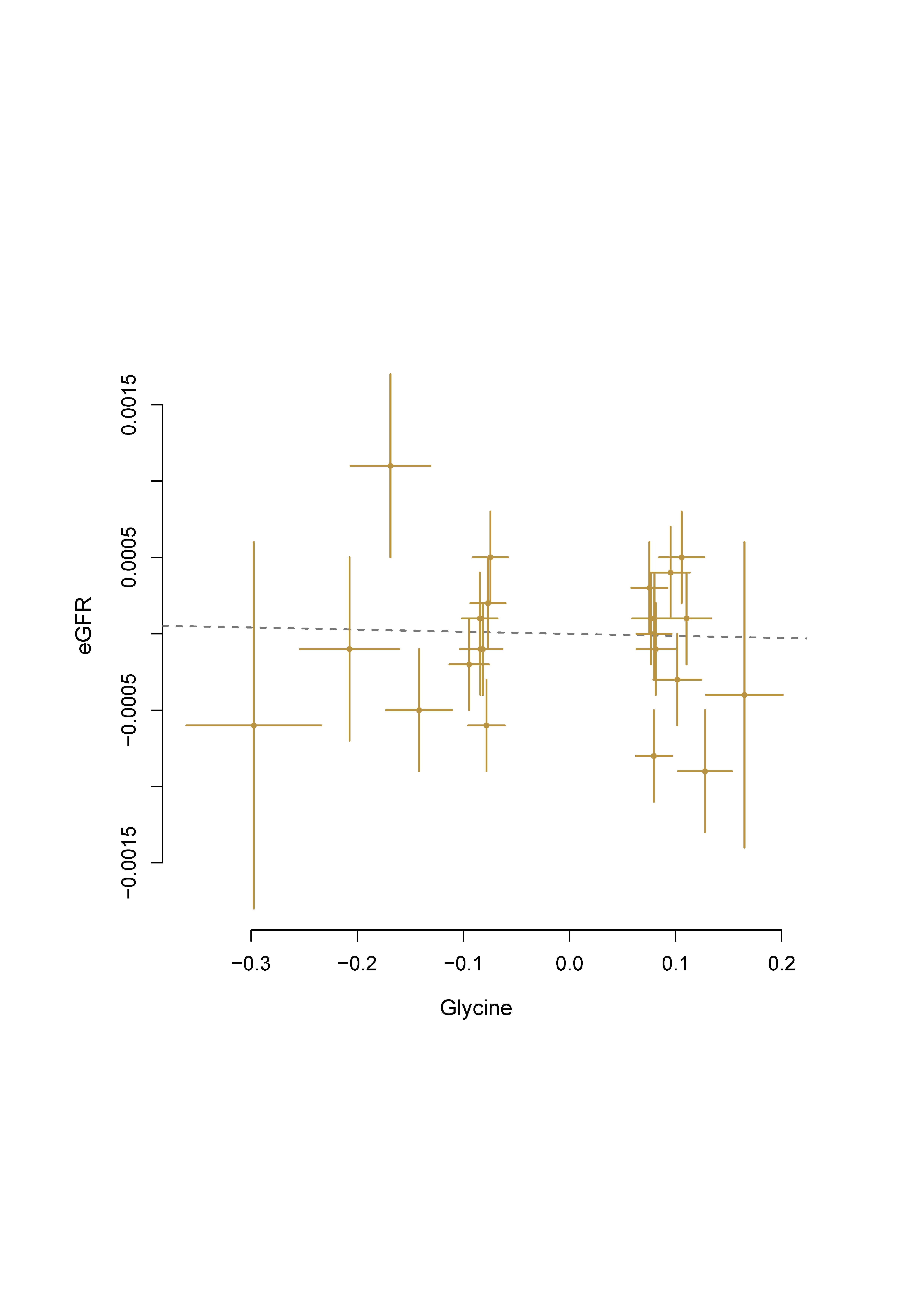

Supplement: LRNF-2024-CS-1772.R2_figure.zip [file IRNF_A_2498090_SM3483.zip › 2E.tif]

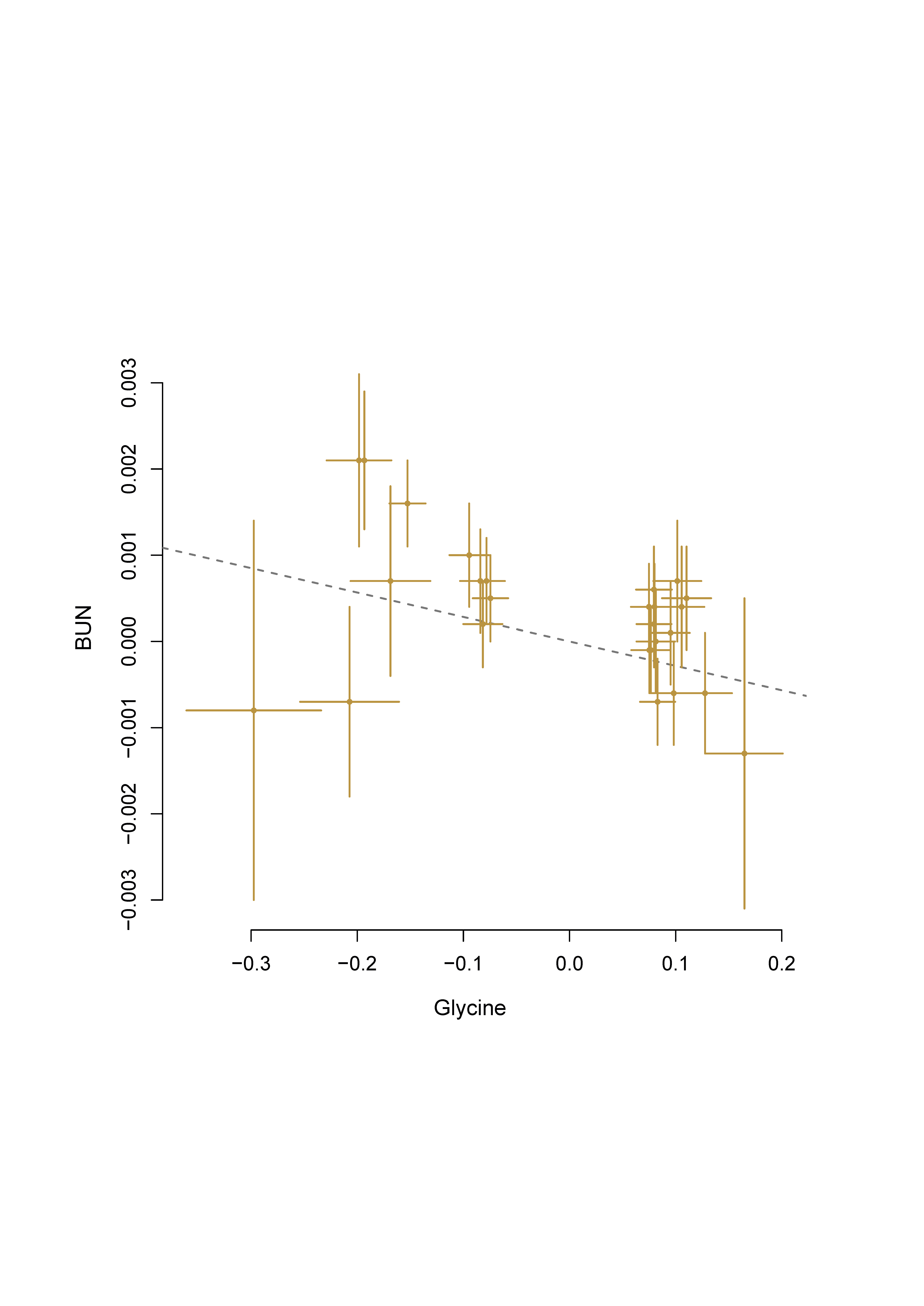

Supplement: LRNF-2024-CS-1772.R2_figure.zip [file IRNF_A_2498090_SM3483.zip › 2F.tiff]

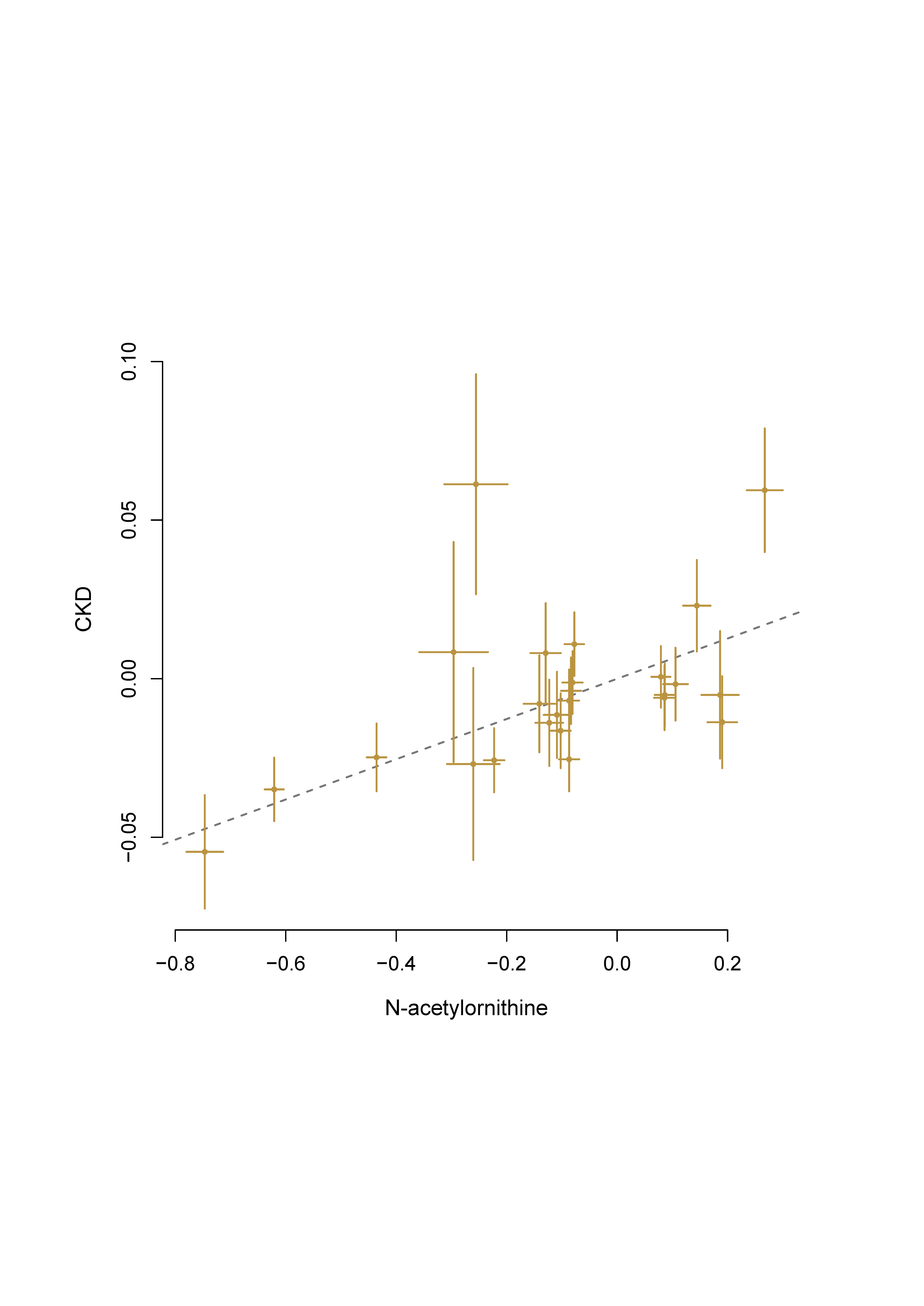

Supplement: LRNF-2024-CS-1772.R2_figure.zip [file IRNF_A_2498090_SM3483.zip › 2G.tif]

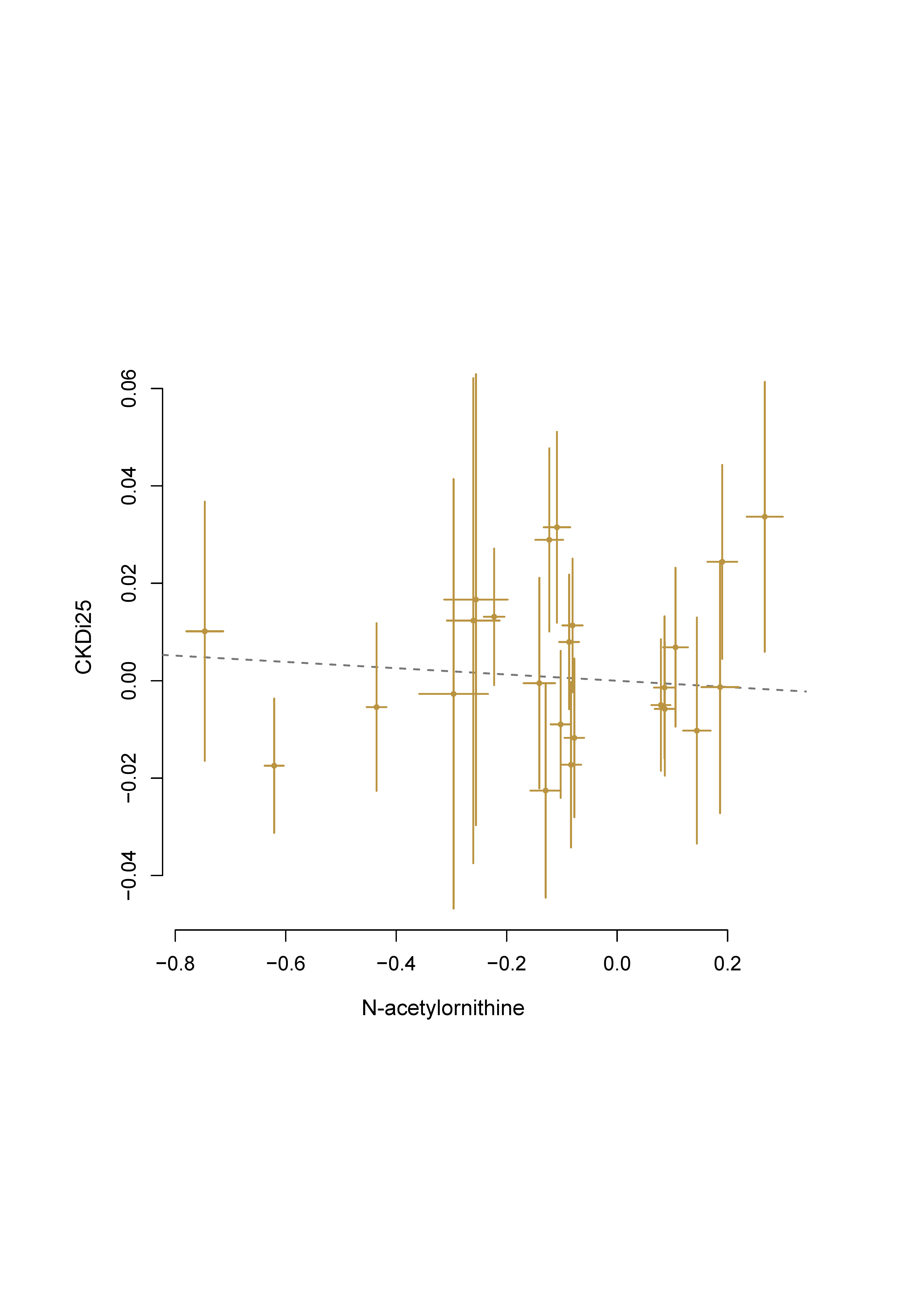

Supplement: LRNF-2024-CS-1772.R2_figure.zip [file IRNF_A_2498090_SM3483.zip › 2H.tif]

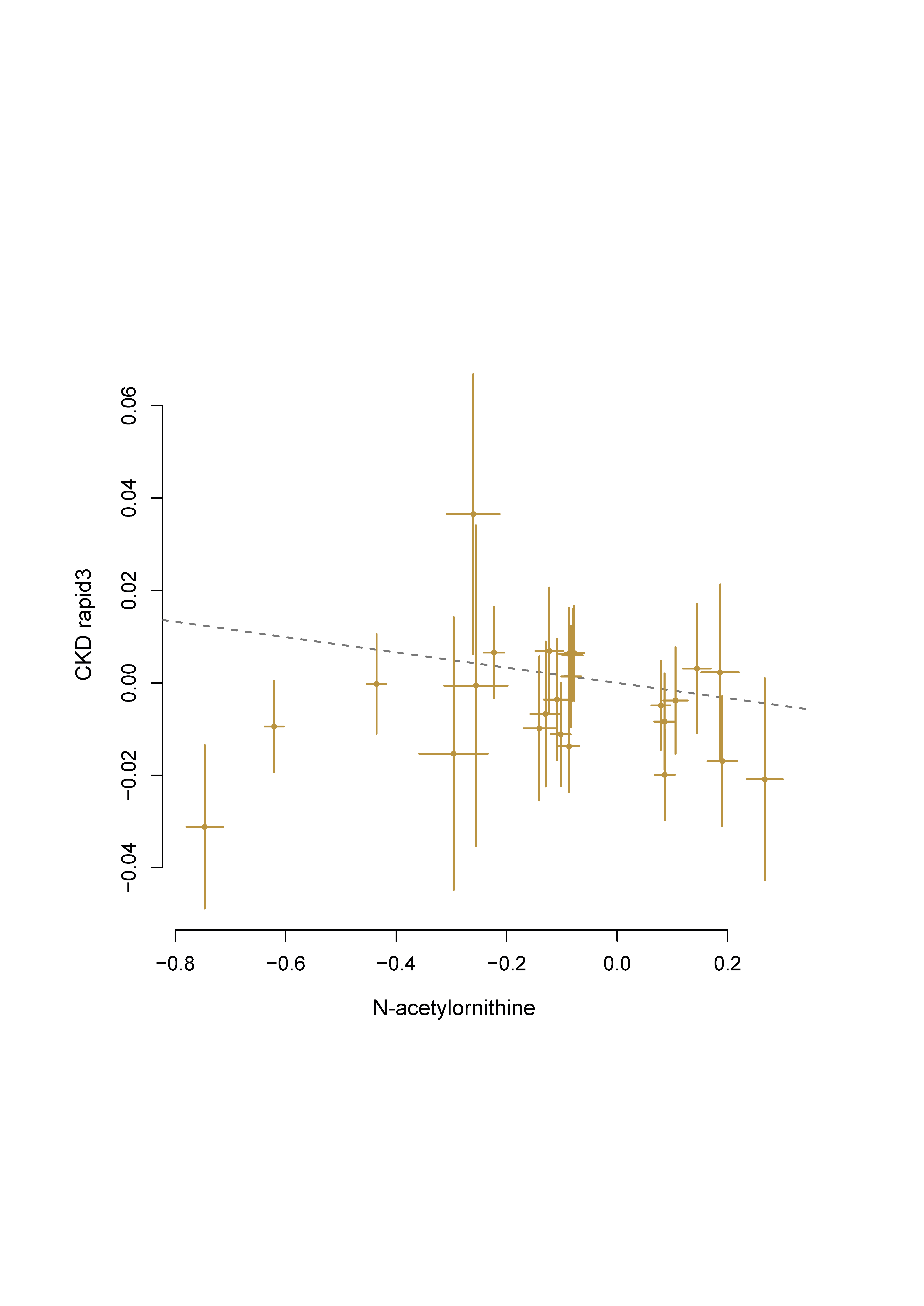

Supplement: LRNF-2024-CS-1772.R2_figure.zip [file IRNF_A_2498090_SM3483.zip › 2I.tif]

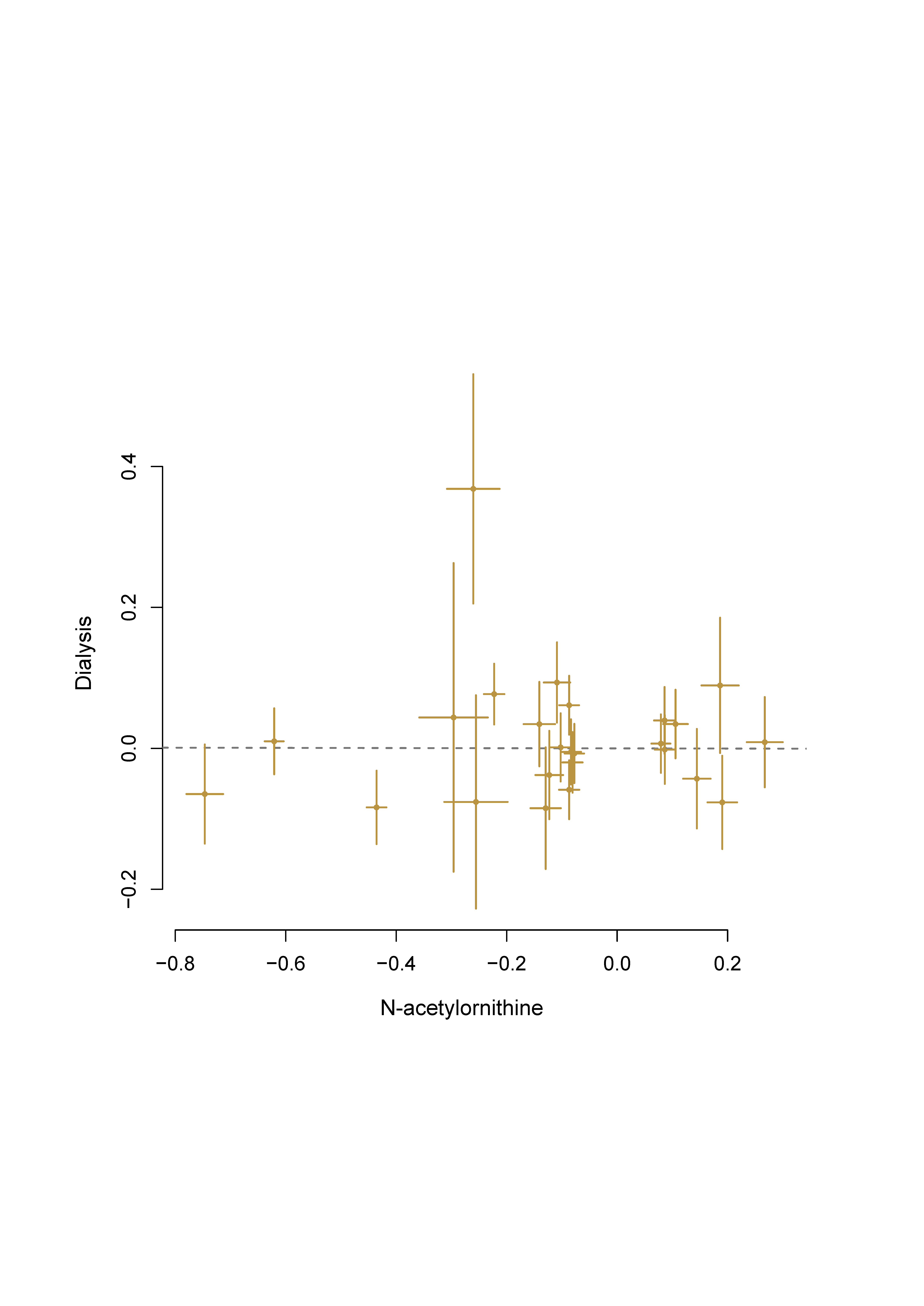

Supplement: LRNF-2024-CS-1772.R2_figure.zip [file IRNF_A_2498090_SM3483.zip › 2J.tif]

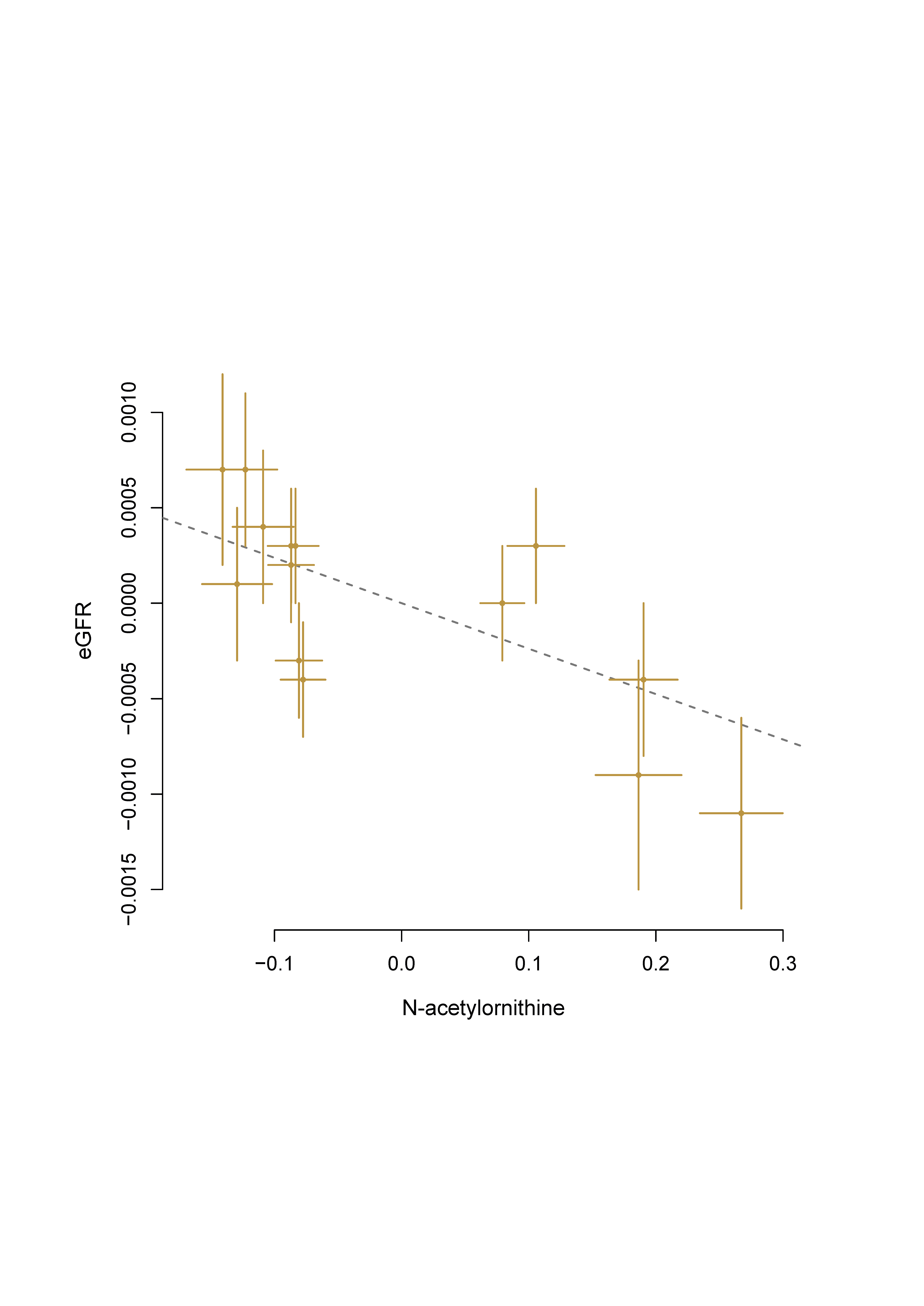

Supplement: LRNF-2024-CS-1772.R2_figure.zip [file IRNF_A_2498090_SM3483.zip › 2K.tif]

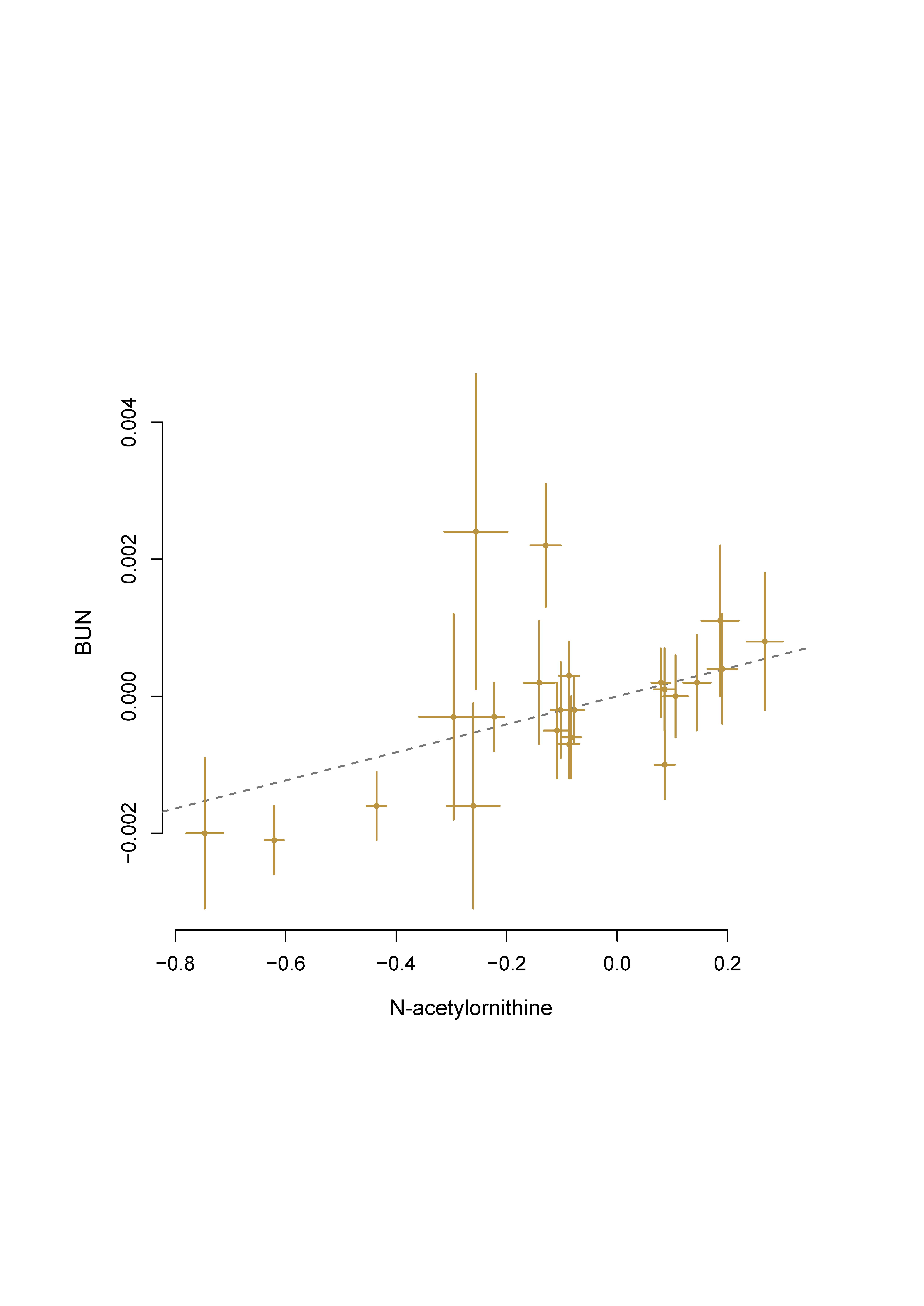

Supplement: LRNF-2024-CS-1772.R2_figure.zip [file IRNF_A_2498090_SM3483.zip › 2L.tiff]

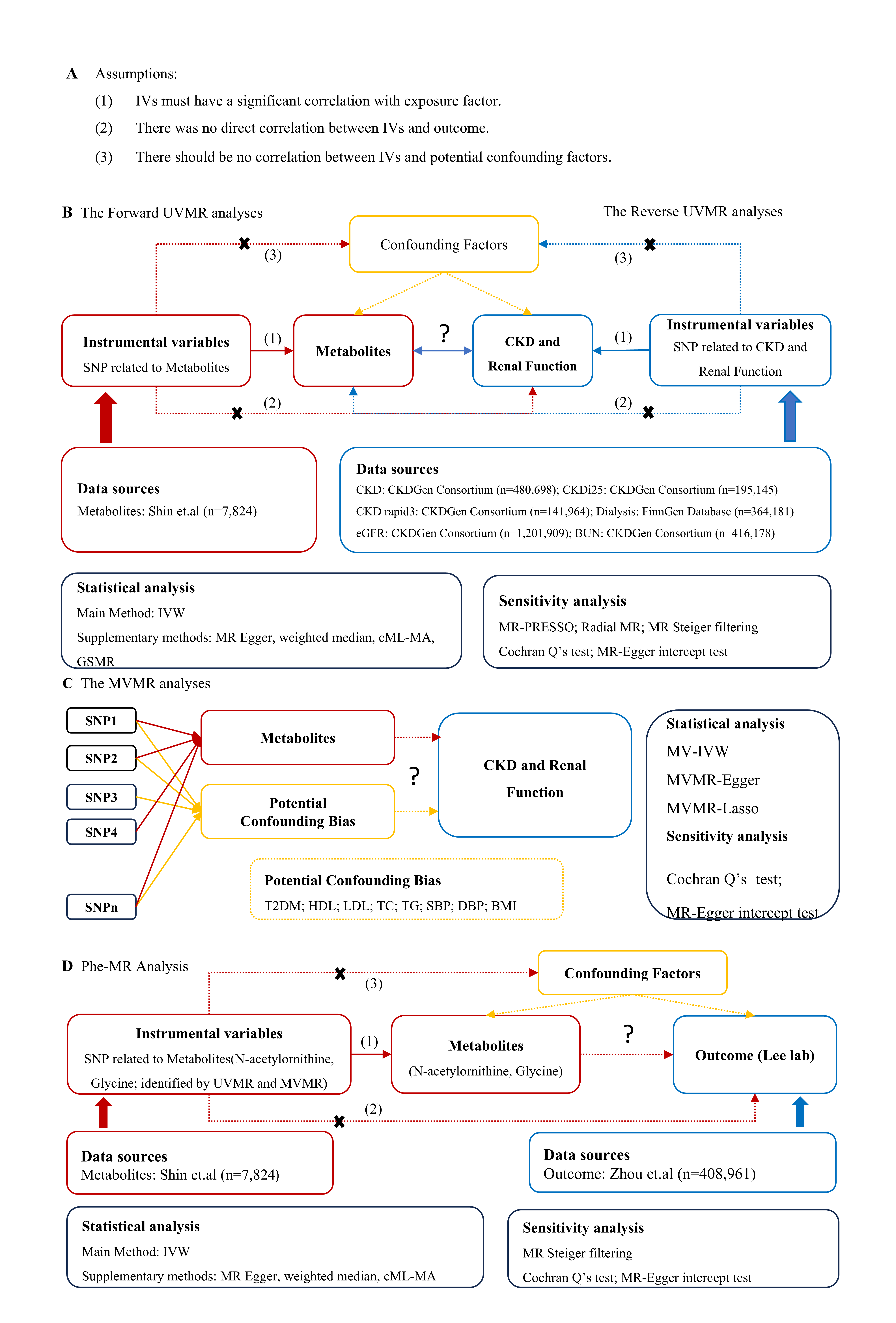

Supplement: LRNF-2024-CS-1772.R2_figure.zip [file IRNF_A_2498090_SM3483.zip › Figure 1.tif]

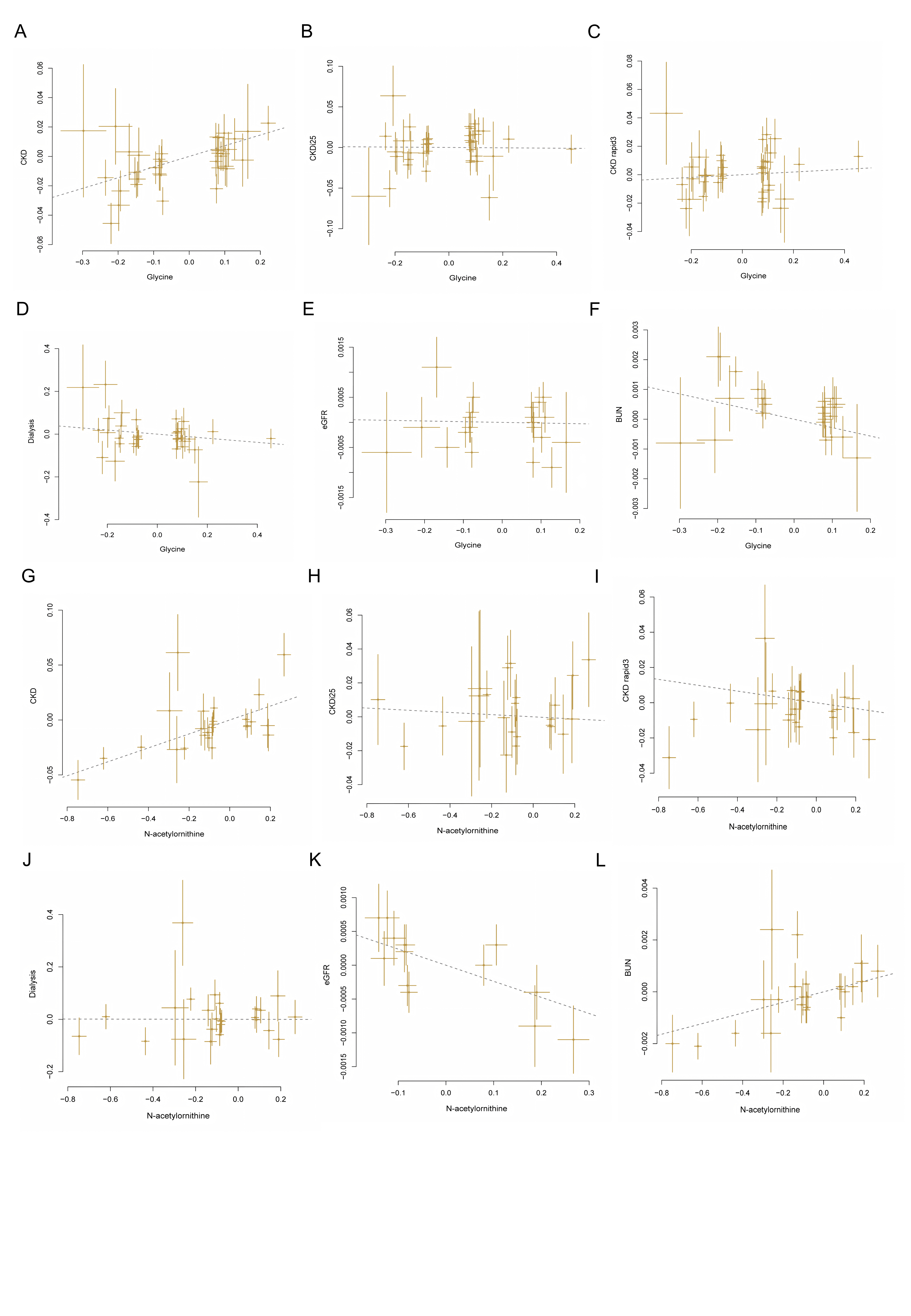

Supplement: LRNF-2024-CS-1772.R2_figure.zip [file IRNF_A_2498090_SM3483.zip › Figure 2.TIF]

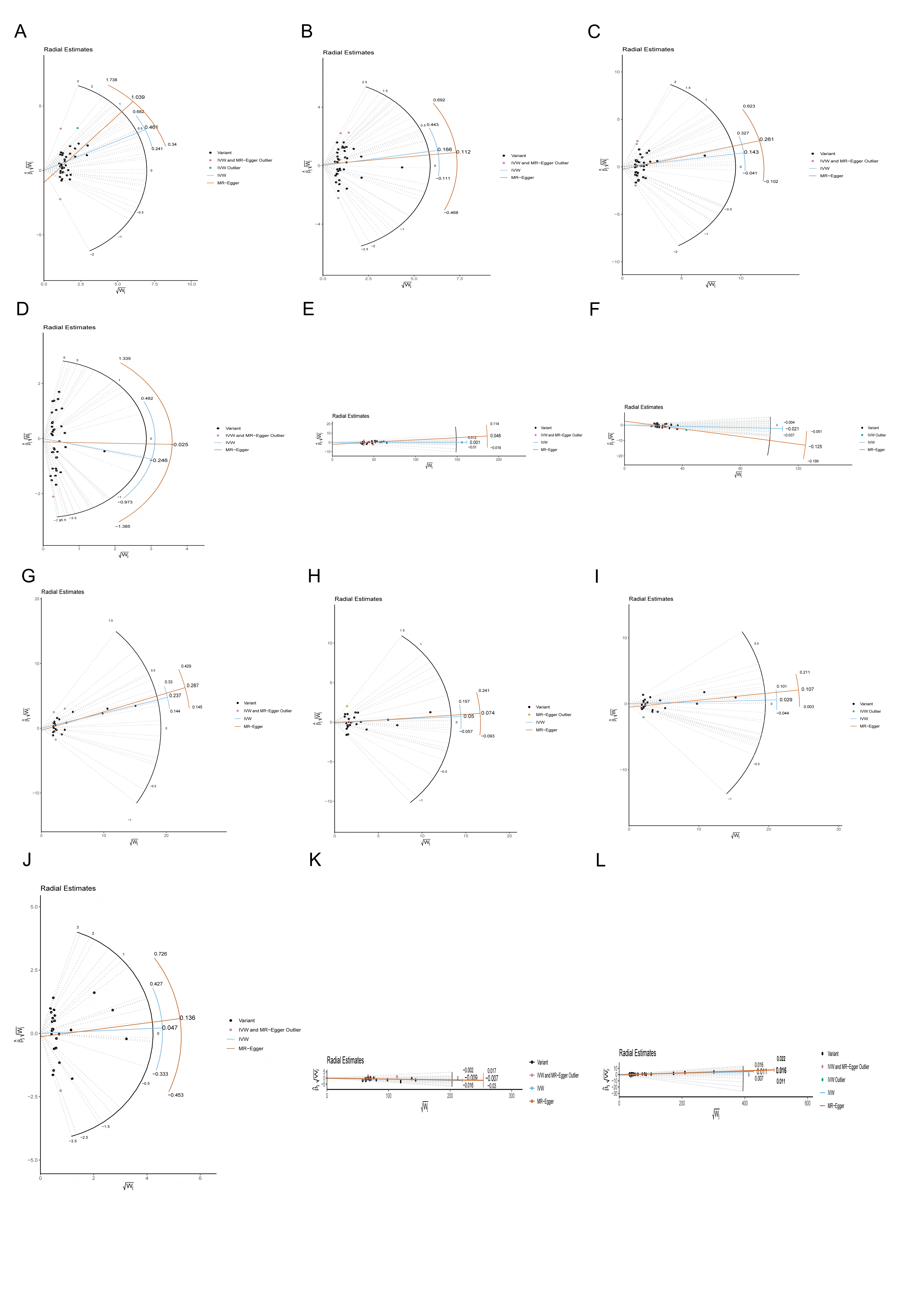

Supplement: LRNF-2024-CS-1772.R2_figure.zip [file IRNF_A_2498090_SM3483.zip › Figure S1.TIF]

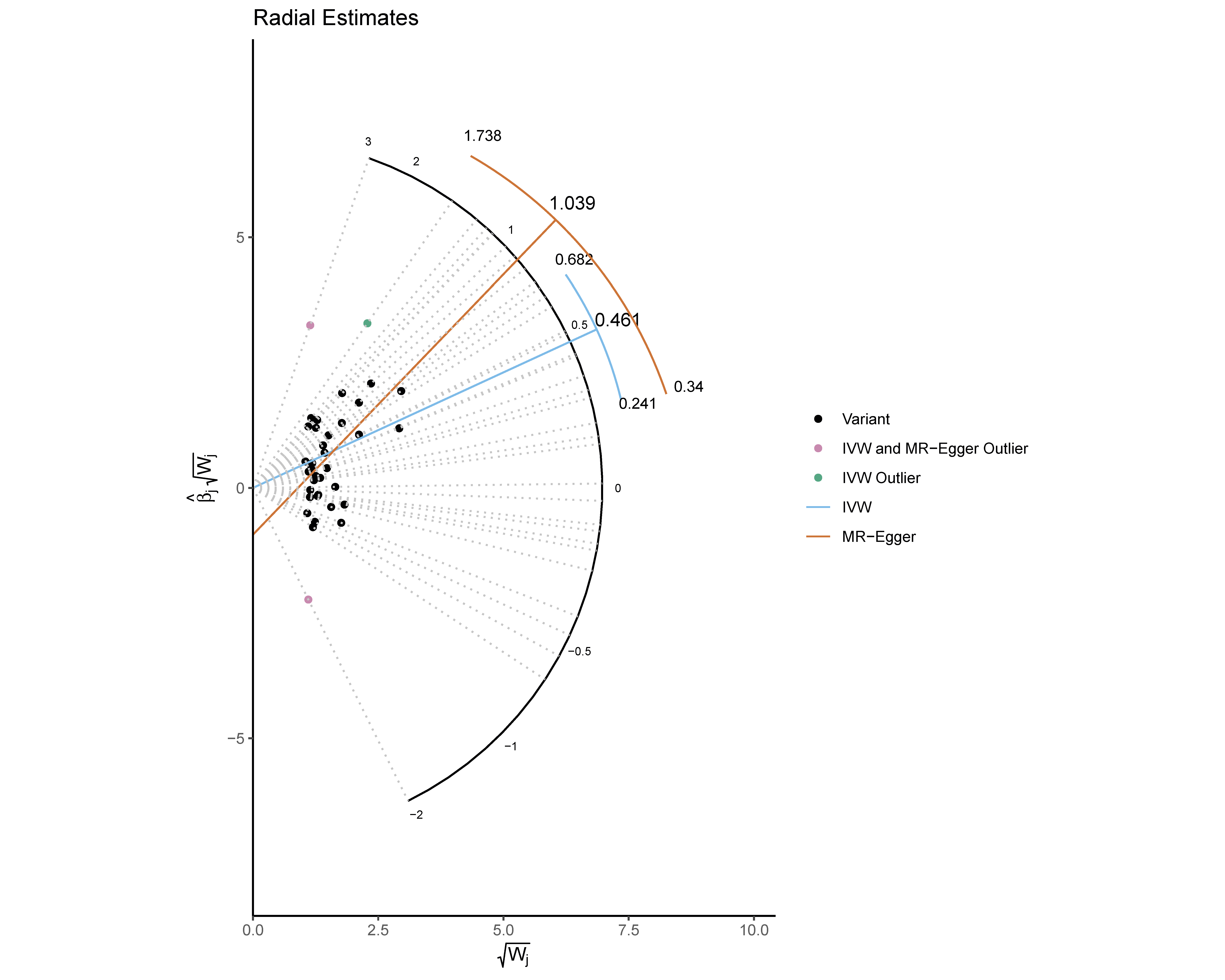

Supplement: LRNF-2024-CS-1772.R2_figure.zip [file IRNF_A_2498090_SM3483.zip › S1A.tif]

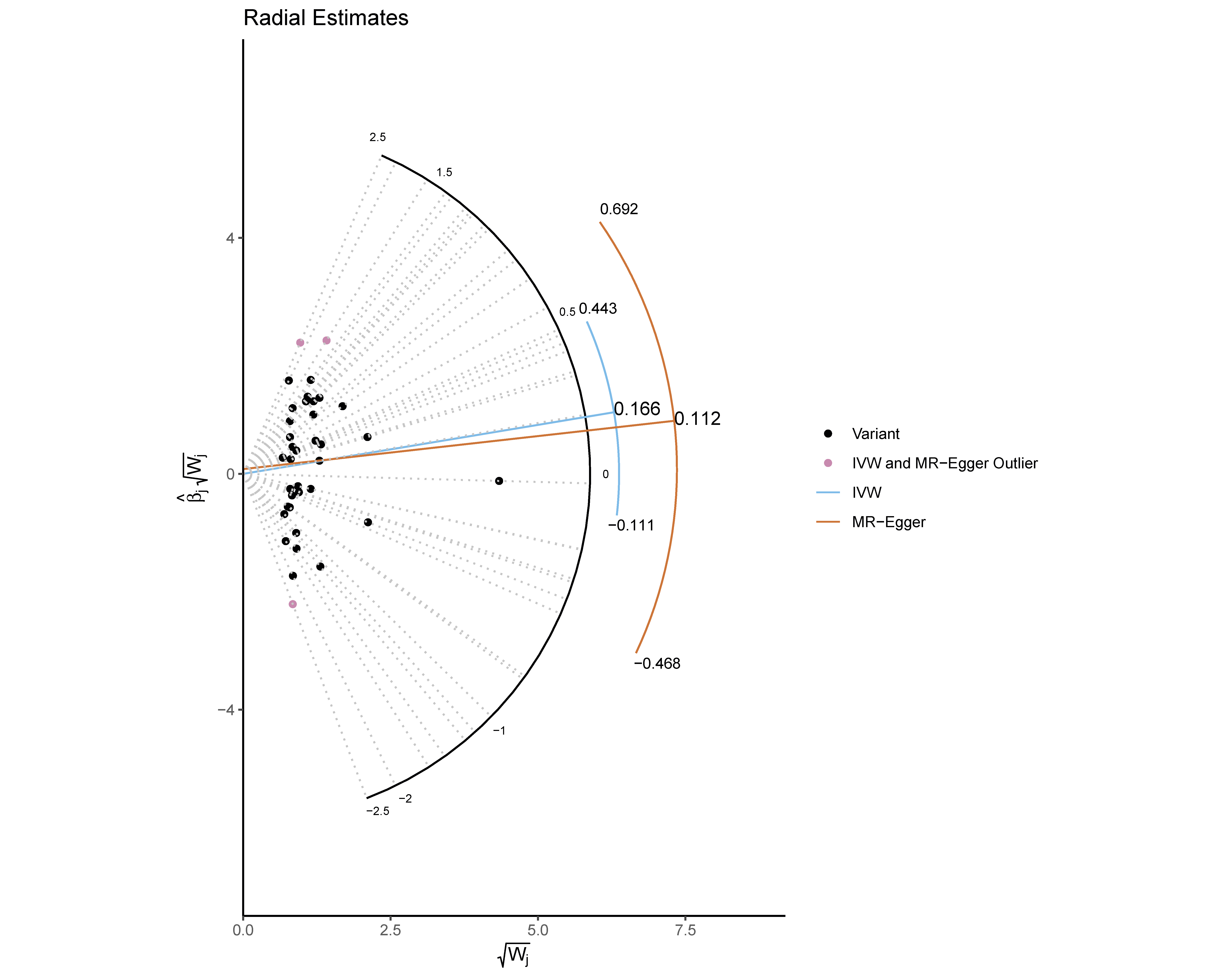

Supplement: LRNF-2024-CS-1772.R2_figure.zip [file IRNF_A_2498090_SM3483.zip › S1B.tif]

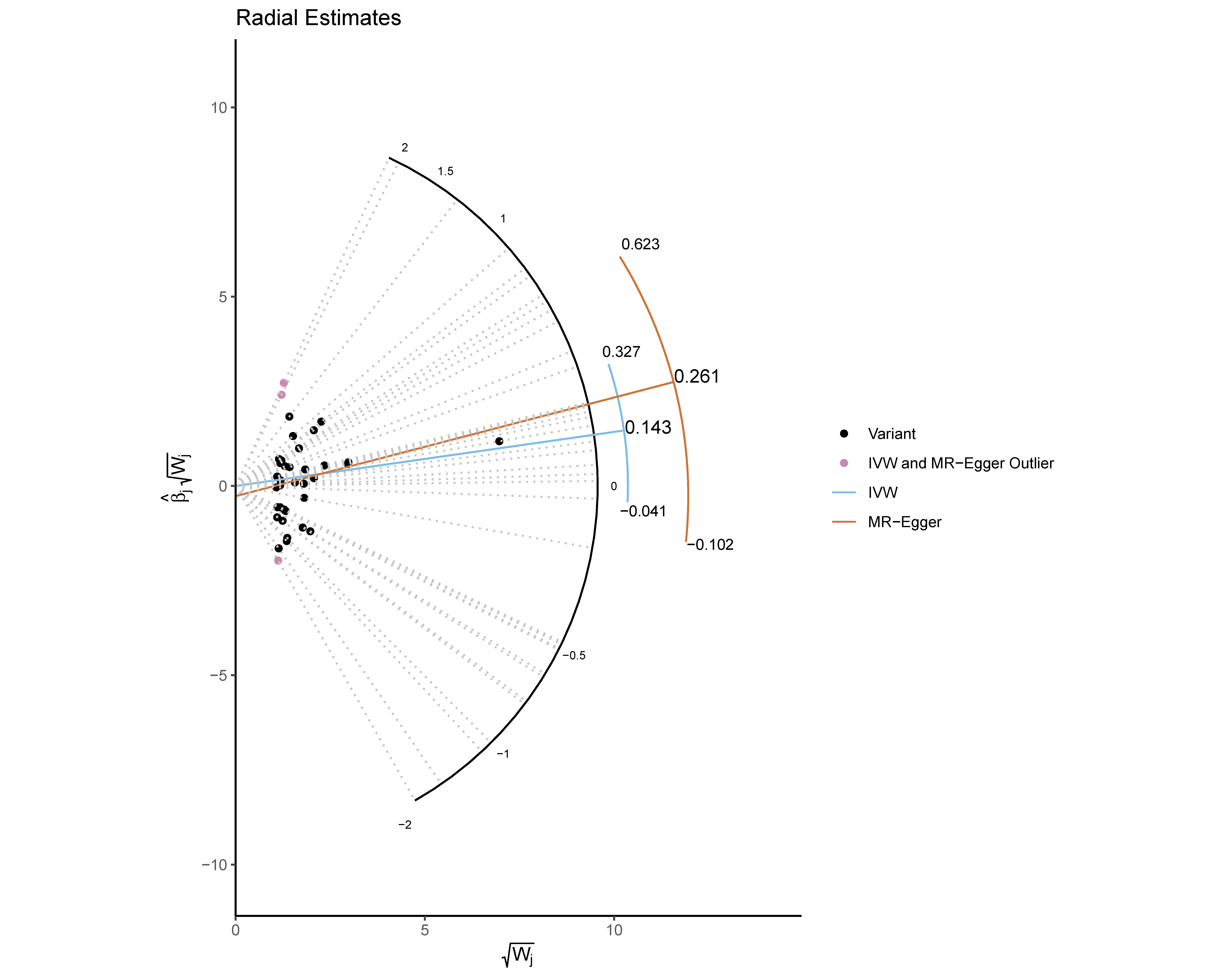

Supplement: LRNF-2024-CS-1772.R2_figure.zip [file IRNF_A_2498090_SM3483.zip › S1C.tif]

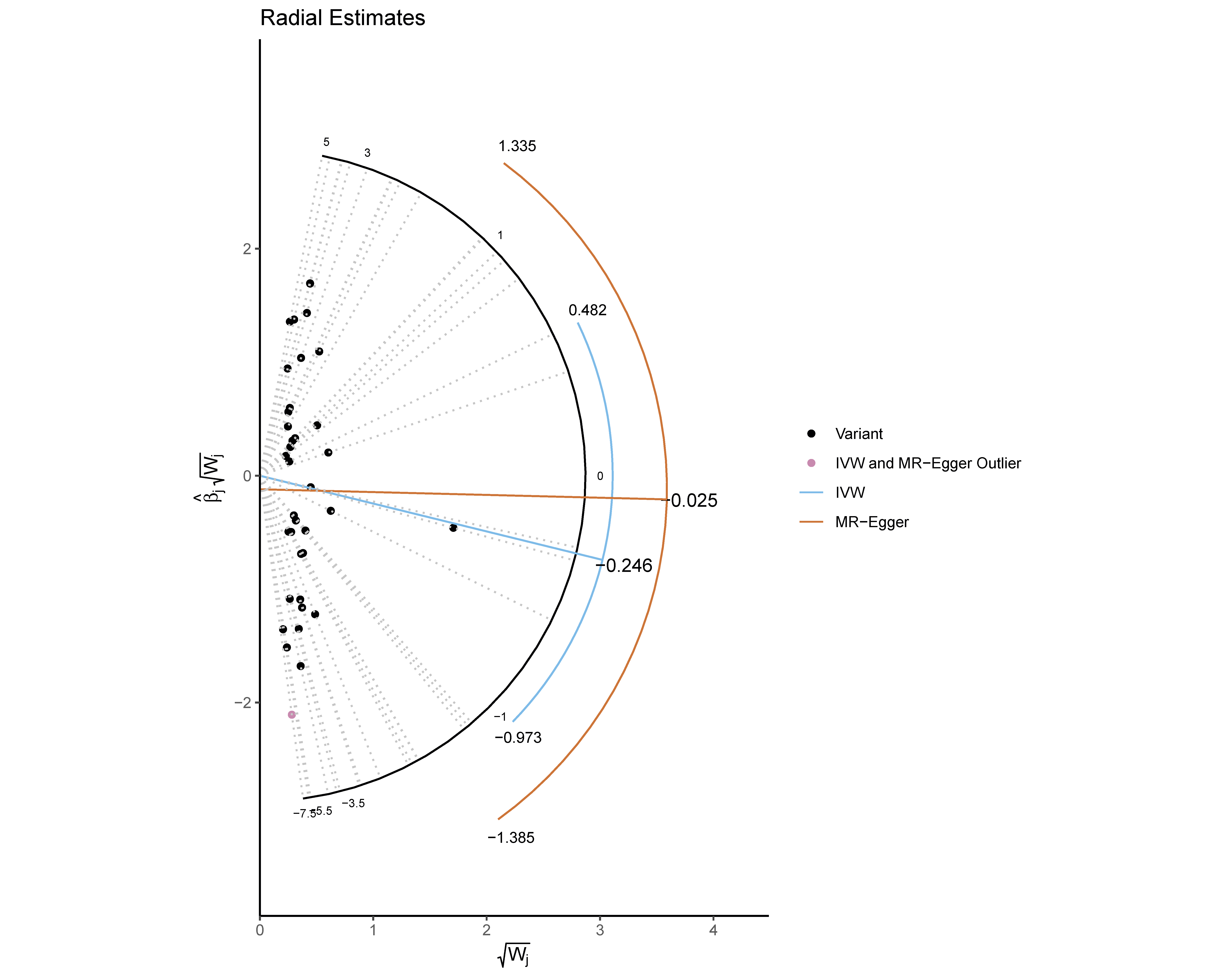

Supplement: LRNF-2024-CS-1772.R2_figure.zip [file IRNF_A_2498090_SM3483.zip › S1D.tif]

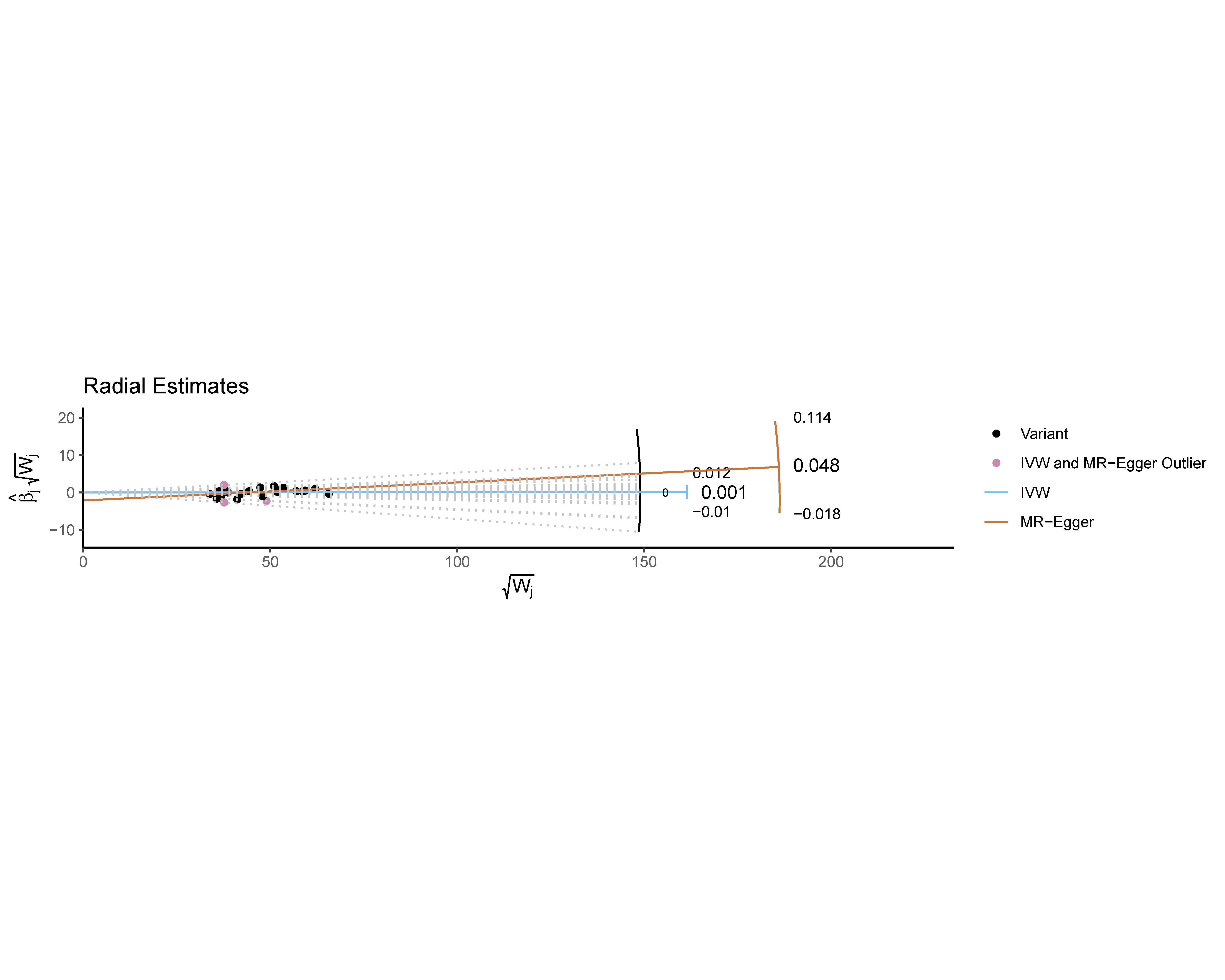

Supplement: LRNF-2024-CS-1772.R2_figure.zip [file IRNF_A_2498090_SM3483.zip › S1E.tif]

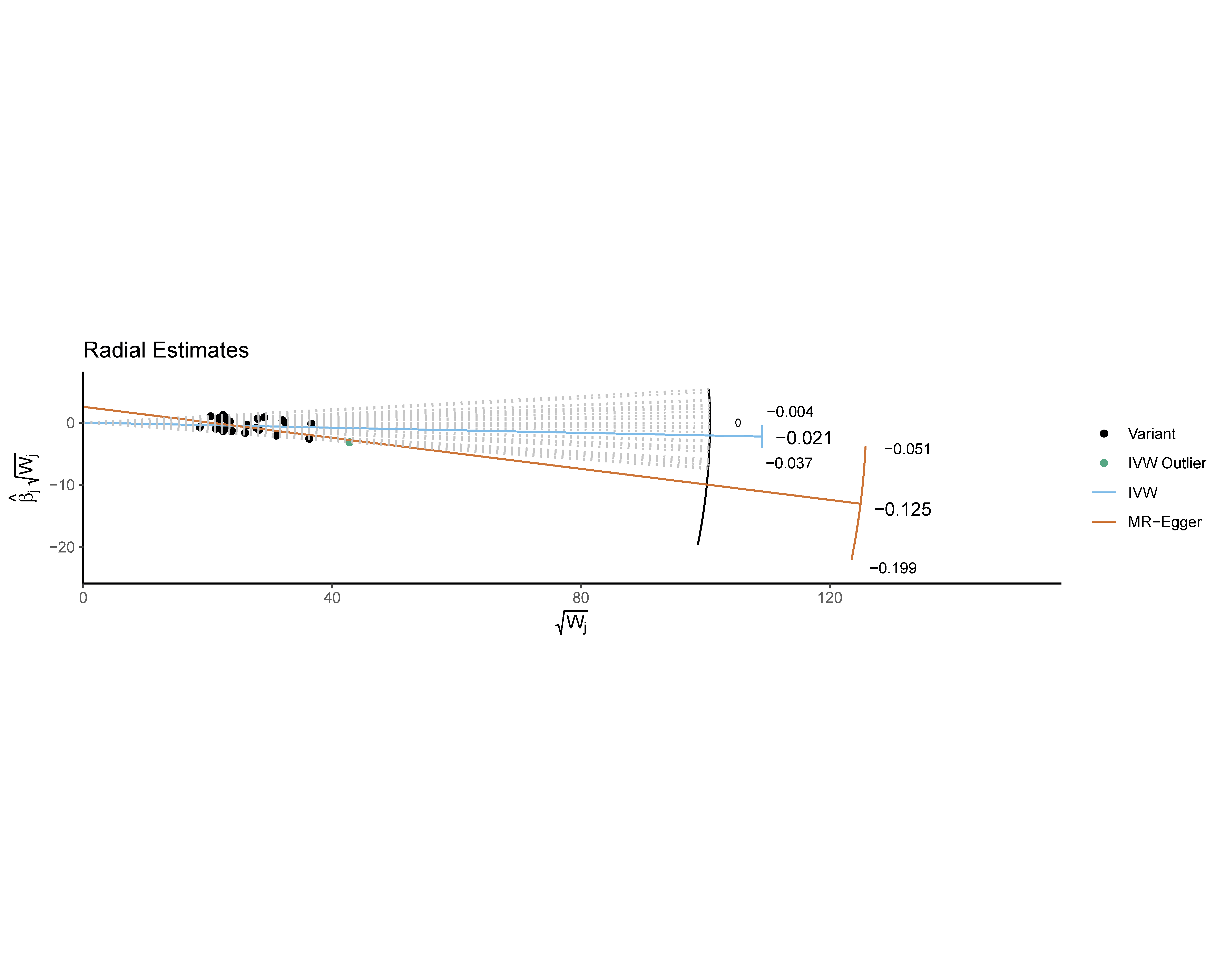

Supplement: LRNF-2024-CS-1772.R2_figure.zip [file IRNF_A_2498090_SM3483.zip › S1F.tif]

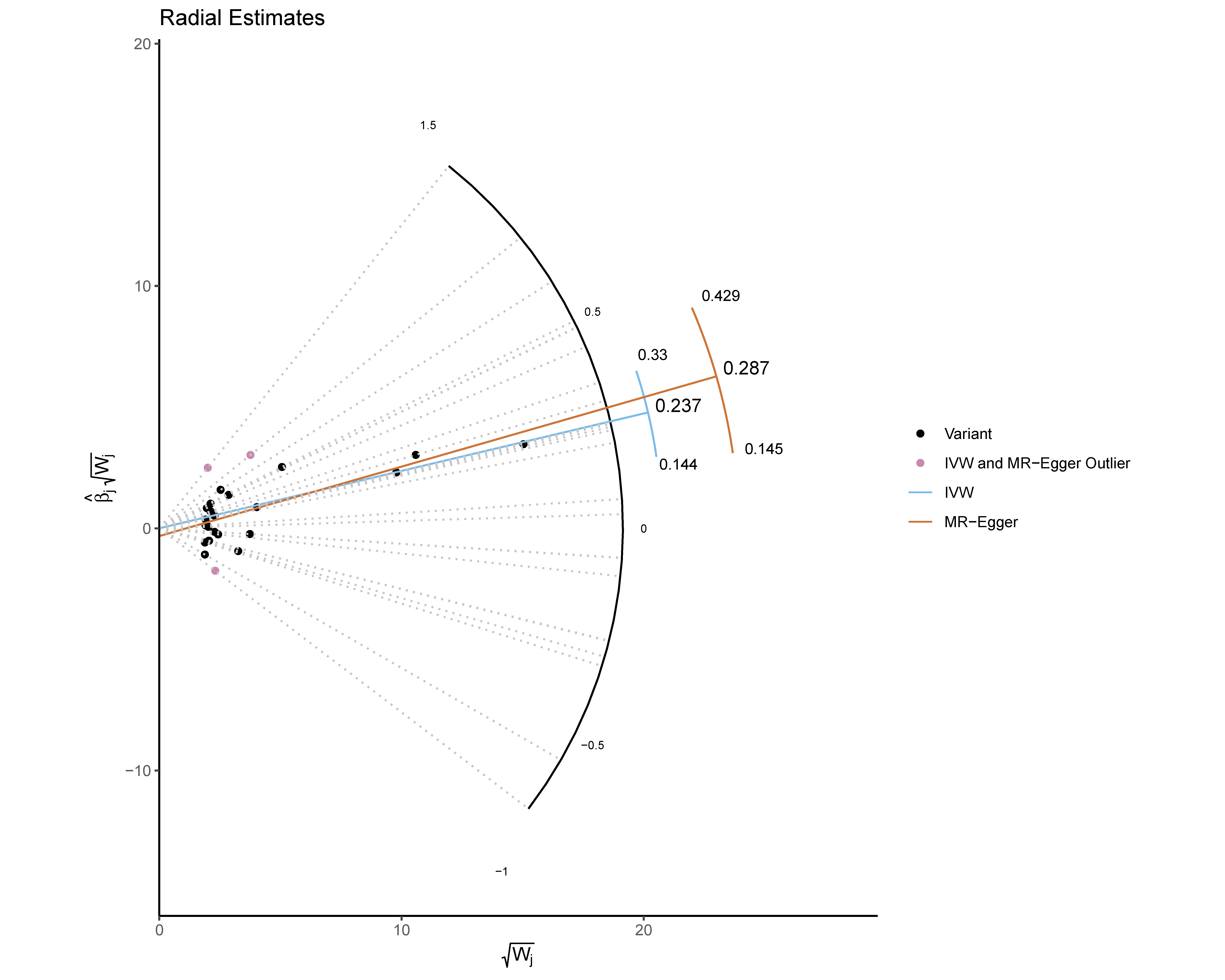

Supplement: LRNF-2024-CS-1772.R2_figure.zip [file IRNF_A_2498090_SM3483.zip › S1G.tif]

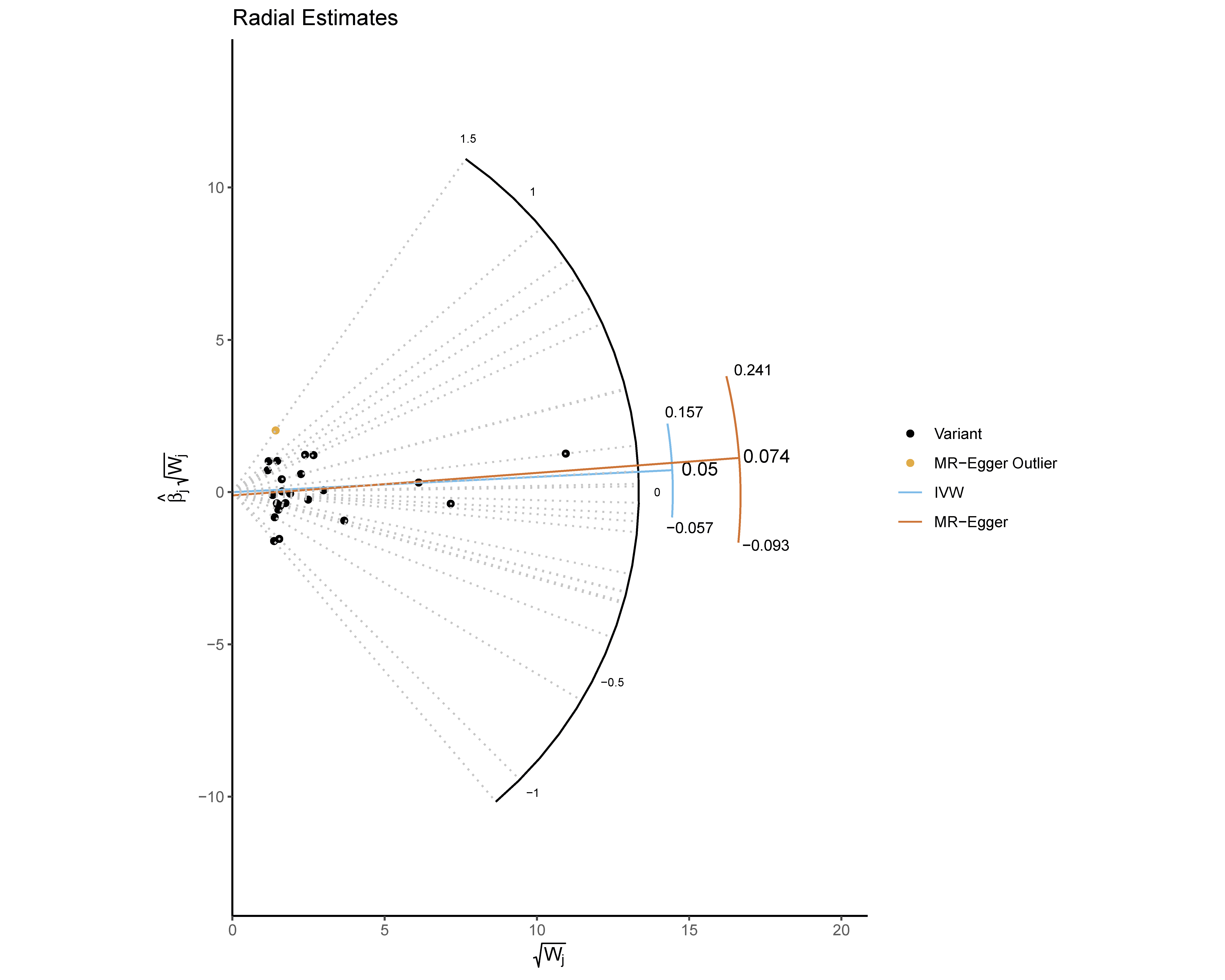

Supplement: LRNF-2024-CS-1772.R2_figure.zip [file IRNF_A_2498090_SM3483.zip › S1H.tif]

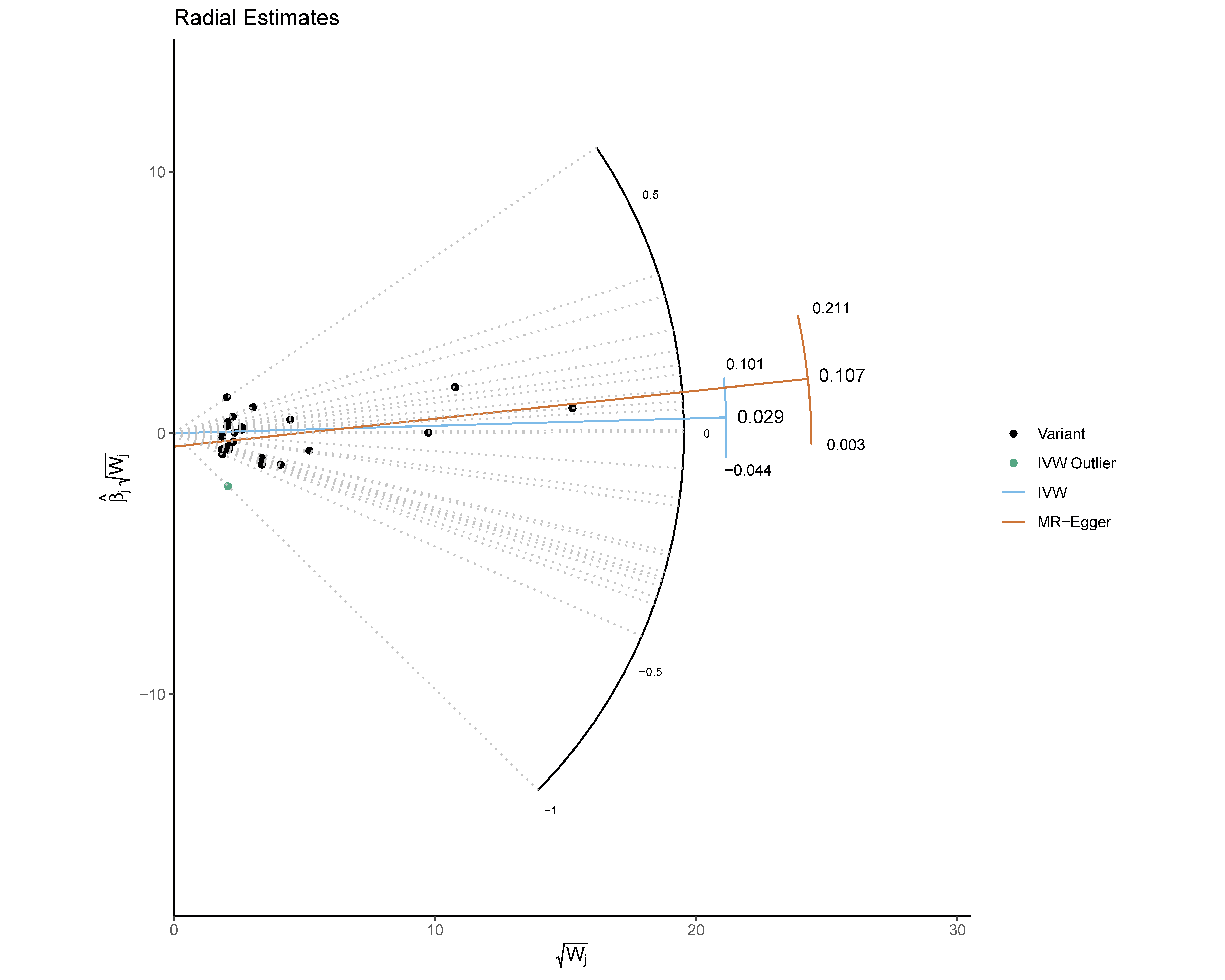

Supplement: LRNF-2024-CS-1772.R2_figure.zip [file IRNF_A_2498090_SM3483.zip › S1I.tif]

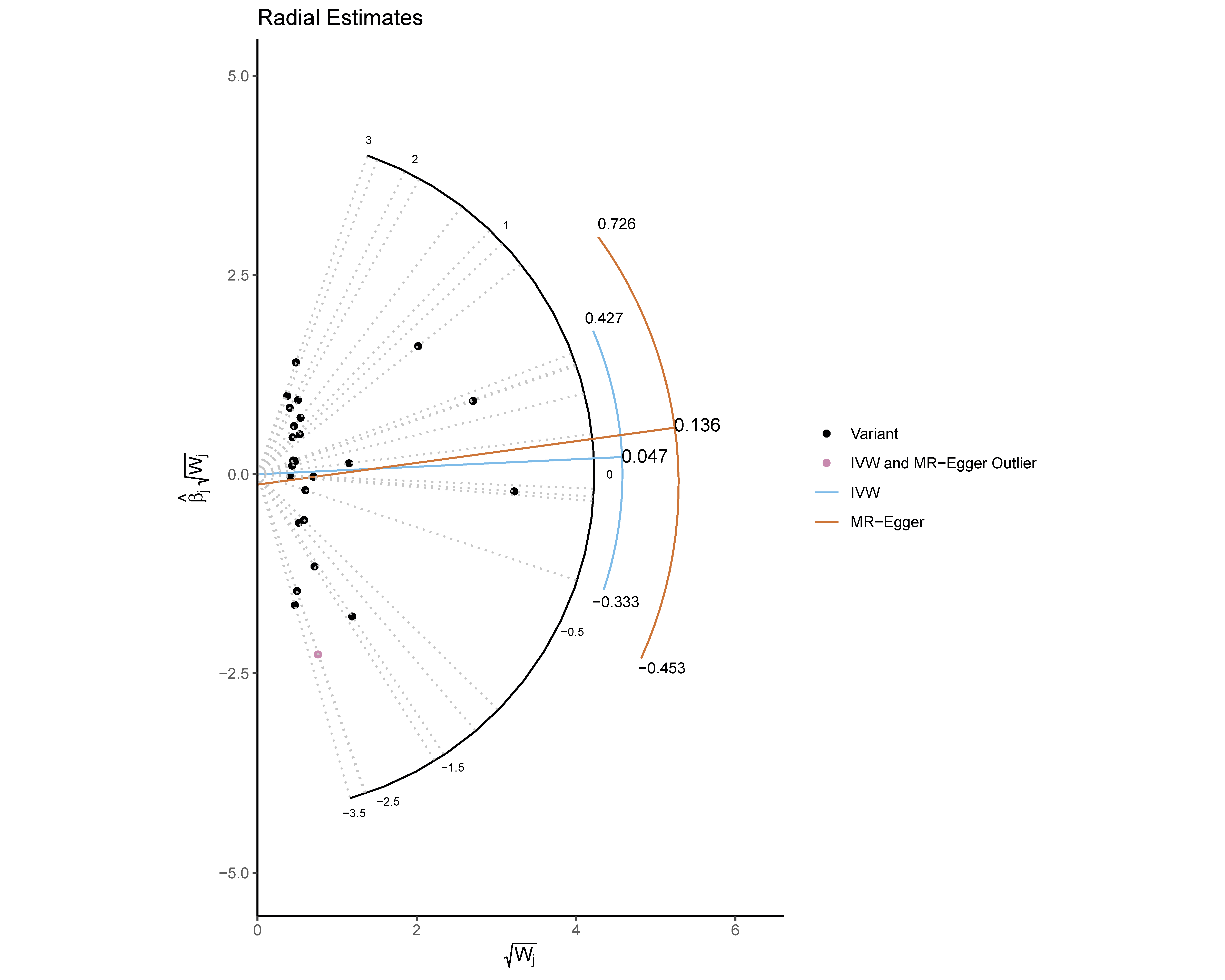

Supplement: LRNF-2024-CS-1772.R2_figure.zip [file IRNF_A_2498090_SM3483.zip › S1J.tif]

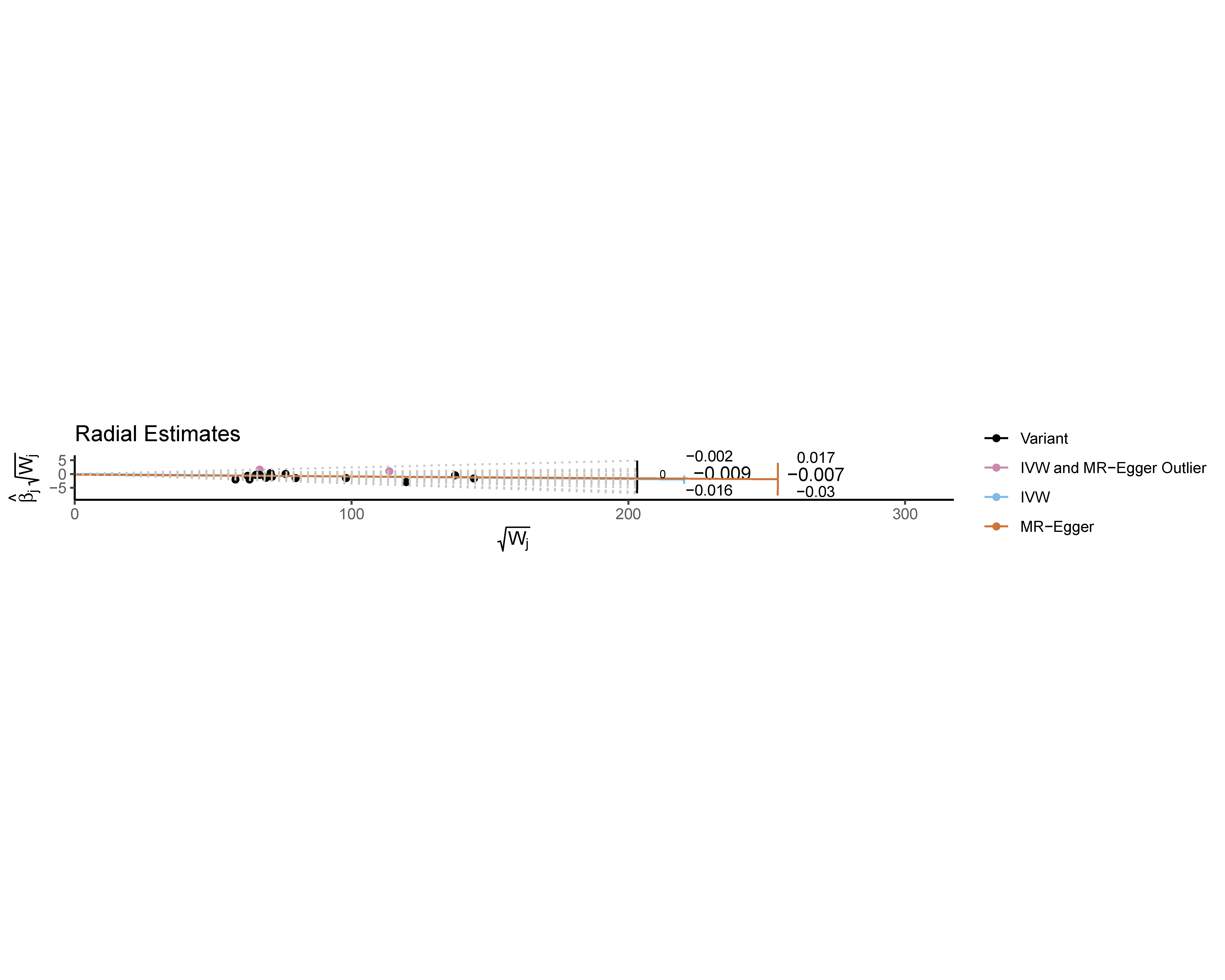

Supplement: LRNF-2024-CS-1772.R2_figure.zip [file IRNF_A_2498090_SM3483.zip › S1K.tiff]

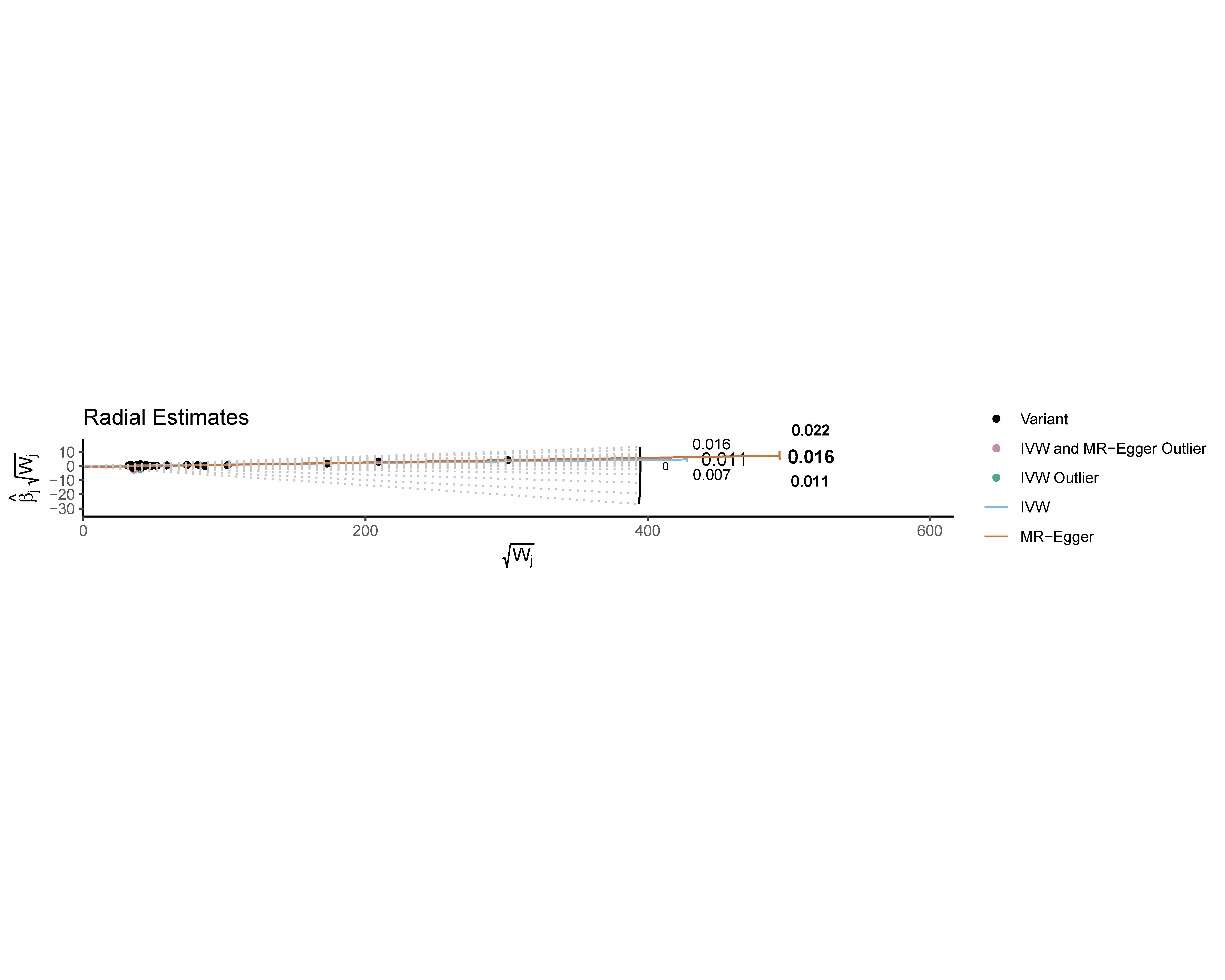

Supplement: LRNF-2024-CS-1772.R2_figure.zip [file IRNF_A_2498090_SM3483.zip › S1L.tiff]
